# Exposome influences: a multi-omics perspective on the combined toxic effects of pharmaceuticals and personal care products in Alzheimer’s disease

**DOI:** 10.3389/ftox.2026.1871830

**Published:** 2026-08-24

**Authors:** İ. İpek Boşgelmez, Beyza Mertaş, Hayrunnisa Deliser

**Affiliations:** 1 Department of Toxicology, Faculty of Pharmacy, Erciyes University, Kayseri, Türkiye; 2 Department of Pharmacology, Faculty of Pharmacy, Düzce University, Düzce, Türkiye

**Keywords:** Alzheimer’s disease, dementia, exposome, multi-omics, personal care products, pharmaceuticals

## Abstract

According to WHO data, approximately 57 million people worldwide were affected by dementia in 2021, with prevalence projected to rise. Alzheimer’s disease (AD), responsible for 60%–80% of dementia cases, continues to be a leading cause of mortality, with current treatments offering limited efficacy and disease-modifying therapies lacking widespread adoption or conclusive safety evidence, shifting the focus toward prevention and risk modification. Risk factors for AD include both non-modifiable elements, such as age, genetics, and gender, and modifiable factors, like environmental pollution, health status, and diet. While age remains the primary non-modifiable risk factor, early-onset dementia represents only up to 9% of cases. Addressing modifiable factors is essential, as it could prevent or delay almost half of dementia cases, with interventions—such as increased physical activity, smoking cessation, alcohol limitation, and overall health management—being significantly associated with a reduced risk. In this context, the exposome approach offers a comprehensive, integrative framework in which both modifiable and non-modifiable risk factors interact to influence individual susceptibility. Within the neural exposome, chronic low-dose exposure to xenobiotics—such as industrial chemicals, pesticides, metals, pharmaceuticals and personal care products (PPCPs), and air pollutants—may induce neurodegeneration via mechanisms including oxidative stress, neuroinflammation, proteinopathies, and epigenetic modifications, although establishing causality remains challenging. Integration of genomics, transcriptomics, proteomics, metabolomics, and lipidomics, combined with artificial intelligence (AI) techniques such as machine learning (ML) and deep learning (DL), provides promising avenues for biomarker discovery, enhanced preventive strategies, early non-invasive diagnosis, and therapeutic target identification by integrating multi-layered biological data with exposure profiles. This review highlights emerging AD risk factors—including PPCPs—underscoring complex, multifactorial nature of AD and exposome, and the requirement for an interdisciplinary research approach, while also addressing several critical research gaps and methodological limitations.

## 1 Introduction

The emerging field of “exposomics” focuses on the “exposome” and employs methods to evaluate both internal and external exposures. The concept of “exposome” which “encompasses life-course environmental exposures, including lifestyle factors, from the prenatal period onwards” was introduced by molecular epidemiologist Christopher Paul Wild in 2005, drawing a parallel with the term “genome” as a methodological analogy, and underscoring the necessity for the development of precise investigative methods to assess environmental exposures that impact human health (Wild, 2005). Rappaport and Smith (2010) proposed defining the “environment” as the body’s internal chemical milieu and “exposures” as the quantities of biologically active chemicals present within this internal environment. In this framework, exposures are not limited to chemicals that enter the body via various routes; they also encompass the internal chemical milieu, which includes xenobiotics as well as chemicals generated by inflammation, oxidative stress, infections, gut microbiota, and other endogenous processes (Rappaport and Smith, 2010). As an amendment, Miller and Jones (2014) described the exposome as “The cumulative measure of environmental influences and associated biological responses throughout the lifespan, including exposures from the environment, diet, behavior, and endogenous processes” (Miller and Jones, 2014), underscoring the essentiality of involvement of a wide range of disciplines in the field. This issue was also raised concerning the etiology of neurodegenerative diseases and dementia: To elucidate causal relationships from environmental correlates, it is essential to integrate multidisciplinary efforts to understand the underlying mechanisms of neurodegeneration and to identify early biological markers (Cavaliere and Gülöksüz, 2022). The importance of exposome lies in the fact that, for many years, the potential health and ecological effects of coincidental chemical mixtures encountered in organisms, wildlife, water, sediments, and soil from various sources have been insufficiently addressed in chemical risk assessment and management, since regulatory risk assessments have mainly focused on evaluating individual substances and chemical products, which may include intentional mixtures or substances with unknown or variable compositions (Backhaus et al., 2025).

**FIGURE 1:**
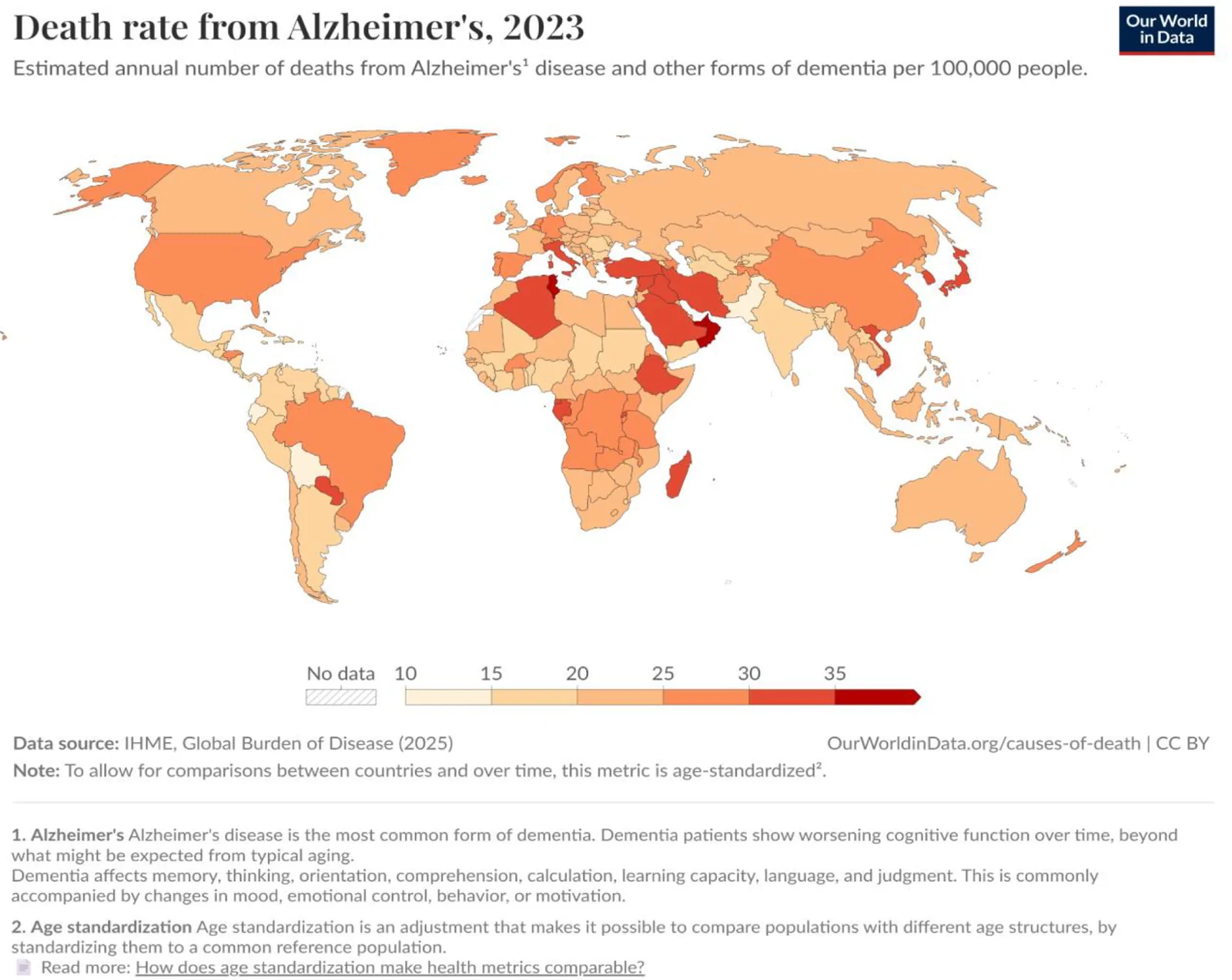
Estimated annual number of deaths from AD and other forms of dementia per 100,000 people (IHME, 2025).

The global prevalence of AD and related dementias (ADRD) is rising, driven by factors such as an aging population, posing significant challenges to healthcare systems worldwide by increasing mortality and disability-adjusted life years (Xiaopeng et al., 2025). Dementia progressively impairs cognitive functions beyond physiological aging, affecting memory, thinking, orientation, comprehension, calculation, learning, language, and judgment, often alongside mood, emotional regulation, behavioral, or motivational changes. According to the World Health Organization (WHO, 2025), in 2021, 57 million people worldwide were living with dementia, with over 60% residing in low- and middle-income countries. Nearly 10 million new cases are diagnosed each year, and it is currently among the leading causes of mortality and a major contributor to disability and dependency among adults 65 and older globally. Dementia arises from various diseases and injuries affecting the brain, with AD being the predominant form, accounting for 60%–70% of cases. In 2019, dementia’s economic impact was valued at US$ 1.3 trillion worldwide, with approximately half of this cost borne by informal caregivers—who provide daily care and supervision (WHO, 2025). The age-standardized estimated annual mortality rate from ADRD per 100,000 individuals in 2023 is illustrated in Figure 1, emphasizing the global significance of this health concern (IHME, 2025). Given that risk factors for AD include both non-modifiable elements, such as age, genetics, and gender, and modifiable factors like environmental pollution, health status, and diet, addressing these modifiable factors is crucial, as it could prevent or delay nearly half of dementia cases. In this context, the exposome approach offers a comprehensive, integrative framework in which both modifiable and non-modifiable risk factors interact to influence individual susceptibility.

In neurodegenerative disorders such as AD and Parkinson’s disease, the concept of “neural exposome” refers to the cumulative toxic influence of a variety of xenobiotics, including industrial chemicals, pesticides, metals and metalloids, as well as ingredients of PPCPs, and other environmental chemicals that may initiate or accelerate disease progression (Chin-Chan et al., 2015; Domingo and Nadal, 2026). The interactions among these toxic agents are a vital aspect; for example, combined exposures can lead to more detrimental consequences than exposure to a single agent (Fu X. et al., 2025; Petrovici et al., 2025). These exposures typically occur through chronic, low-dose exposures, generally with synergistic interactions, rather than through acute or high-dose exposures to a single toxicant (Tang, 2020; Domingo and Nadal, 2026; Fang et al., 2026). Such factors are often implicated in mitochondrial impairment, sustained oxidative stress, and the deposition of neurotoxic proteins, including amyloid beta (Aβ) and tau, notably in AD patients with genetic predispositions.

In this review, we sought to summarize how exposure to various xenobiotics within the neural exposome—such as industrial chemicals and PPCPs—may contribute to neurodegeneration through mechanisms like oxidative stress, neuroinflammation, and proteinopathies. In addition, the importance of interdisciplinary research and advanced analytical tools, such as multi-omics, bioinformatics, and AI, to discover biomarkers, improve prevention, enable early diagnosis, and identify therapeutic targets, while also highlighting emerging risk factors, including medications and PCPs, and addressing current research gaps and methodological challenges, was briefly evaluated. This narrative review was based on a structured literature search of PubMed, Scopus, and Web of Science to identify peer-reviewed publications relevant to the abovementioned objectives, published between January 2000 and May 2026. The search strategy combined Medical Subject Headings (MeSH), where applicable, with free-text keywords and Boolean-operators, and was tailored to indexing conventions of each database. Search terms included, “Alzheimer’s disease (AD)” “dementia,” “environmental exposure,” “chemical mixtures,” “exposome,” “multi-omics,” “omic,” “exposomics,” “genomics,” “transcriptomics,” “proteomics,” “metabolomics,” “epigenomics,” “epigenetics,” “lipidomics,” “pharmaceuticals,” “personal care products (PCPs),” “pharmaceuticals and personal care products (PPCPs),” “pesticides,” “metals,” “microplastics,” “endocrine-disrupting chemicals (EDCs),” “neurotoxicity,” “neurodegeneration,” and related terms. Foundational studies, recent review articles, and original experimental, epidemiological, and clinical investigations that substantially advanced understanding of the topic were prioritized. To ensure comprehensive coverage of the literature, additional publications were identified through manual screening of references from key reviews and original research articles, as well as backward and forward citation tracking. Selected studies were evaluated for their relevance to objectives, with particular emphasis on mechanistic, toxicological, epidemiological, clinical, and multi-omics evidence regarding the roles of pharmaceuticals, PCPs, and other environmental exposures in AD and related neurodegenerative processes. Publications lacking clear mechanistic relevance or providing limited information on exposure–disease relationships were not further evaluated.

## 2 Exposome and the mixtures problem: synergistic toxicity

In daily life, individuals are concurrently exposed to multiple chemicals at low-doses via diverse sources, including PPCPs. These mixture exposures may result in synergistic biological effects that exceed those anticipated from individual substances (Constantinescu et al., 2025). In the context of AD, the mixtures problem caused by multiple chemical exposures such as PPCPs can be explained through common biological pathways that overlap with the main pathological axes specific to the disease (Aβ, tau and mitochondrial hypotheses): Aβ neurotoxicity, tau neurotoxicity and mitochondrial dysfunction in AD from an early stage, synergistically reinforcing oxidative/nitro-oxidative stress, neuroinflammation, bioenergetics it triggers deterioration and apoptosis (Fišar, 2022). An age-related rise in reactive oxygen species (ROS), mitochondrial dysfunction, and metal homeostasis perturbation, which is further exacerbated by exposure to various environmental stressors (such as pesticides, metals, and other xenobiotics), is strongly correlated with Aβ deposition, hyperphosphorylated tau, synaptic failure, and memory loss (Ashok et al., 2015; Teleanu et al., 2022). In animal and cellular models, co-administration of Aβ with metals or pro-oxidant mixtures results in more severe mitochondrial damage, disrupted biochemical energy production, and hippocampal neurodegeneration compared with Aβ alone, empirically supporting the synergistic relationship between Aβ and oxidative stress (Karapetyan et al., 2022).

From an AD perspective, the mixture problem can be evaluated both in terms of frequently co-occurring chemical combinations and in terms of regulatory frameworks that do not adequately reflect this reality. A comprehensive survey of consumer products demonstrated that the most commonly co-occurring triple combination in PCPs and cleaning products is 2-phenoxyethanol + methylparaben + ethylparaben, found together in 1,059 different products. The same analysis also reported that paraben–glycol ether and paraben fragrance component combinations are highly prevalent, indicating that consumers are exposed to multi-component “cocktails” even through a single product (Gabb and Blake, 2016). Human studies indicate that exposure to mixtures of parabens and/or triclosan induces oxidative stress; however, findings on neurocognitive effects remain inconsistent and generally weak. Although some attention and cognitive changes have been observed in older adults and children, these results have not been statistically significant (Ren X. et al., 2023; Oskar et al., 2024). A comprehensive bioinformatics and molecular docking analysis identified APP, DRD2, and SLC6A3 as reliable predictive biomarkers and indicated that exposure to triclosan may increase the risk of AD by reducing neuroprotective APP expression and interfering with dopaminergic neurotransmission, given the high affinity of triclosan for DRD2 (Cheng et al., 2025). Particularly in terms of AD risk, the long-term, low-dose, cumulative neurological effects of these commonly used PCPs remain an open area for investigation. Current risk assessment frameworks are largely based on a single-chemical–single-endpoint paradigm. However, in real life, individuals are exposed to hundreds of chemical compounds through food, water, and PCPs. Accordingly, data on the risks associated with low-dose, near-threshold mixture exposures, especially in terms of neurotoxicity and neurodegeneration, remain very limited (Elcombe et al., 2022). The exposome approach can help identify distinct chemical signatures and mixture profiles associated with AD and related disorders by analyzing numerous chemical features alongside genomic data.

## 3 AD risk factors and the role of exposome

**FIGURE 2:**
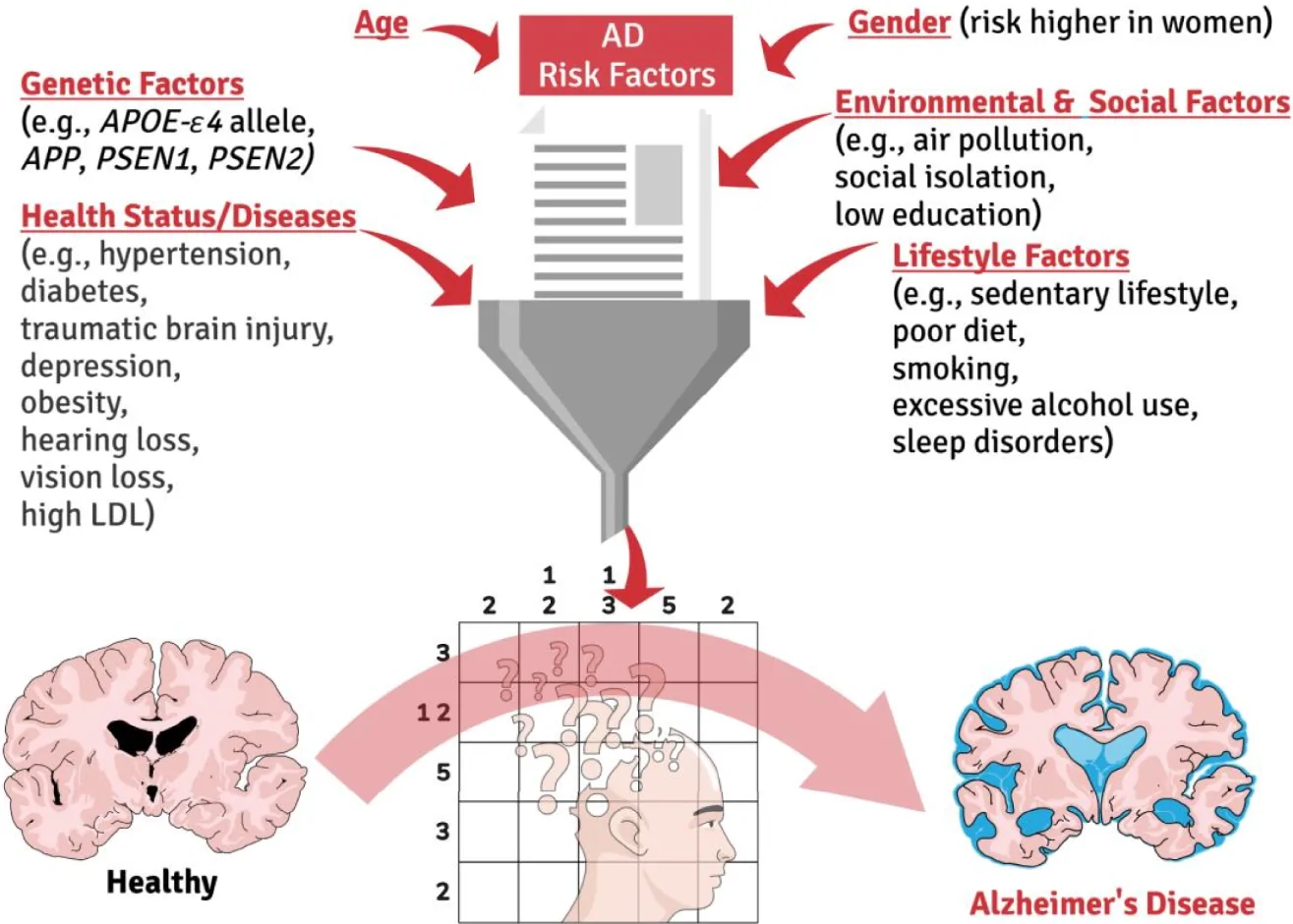
Currently known AD risk factors.

Currently known risk factors for AD (Figure 2) comprise non-modifiable and potentially modifiable risk factors reported by the Lancet Commission (Livingston et al., 2024), which, if addressed at different stages during the life course, could prevent or delay nearly 45% of dementia cases. Although age is the primary non-modifiable risk factor for dementia, it is not an inevitable consequence of biological aging. Evidence shows that risk can be reduced through regular physical activity, smoking cessation, moderate alcohol intake, maintaining a healthy weight, a balanced diet, and managing blood pressure, cholesterol, and blood glucose. Other risk factors include depression, social isolation, low education, cognitive inactivity, and air pollution. Moreover, women are disproportionately affected by dementia, either experiencing higher disability-adjusted life years and mortality rates or providing 70% of the caregiving hours for individuals with dementia (WHO, 2025).

**FIGURE 3:**
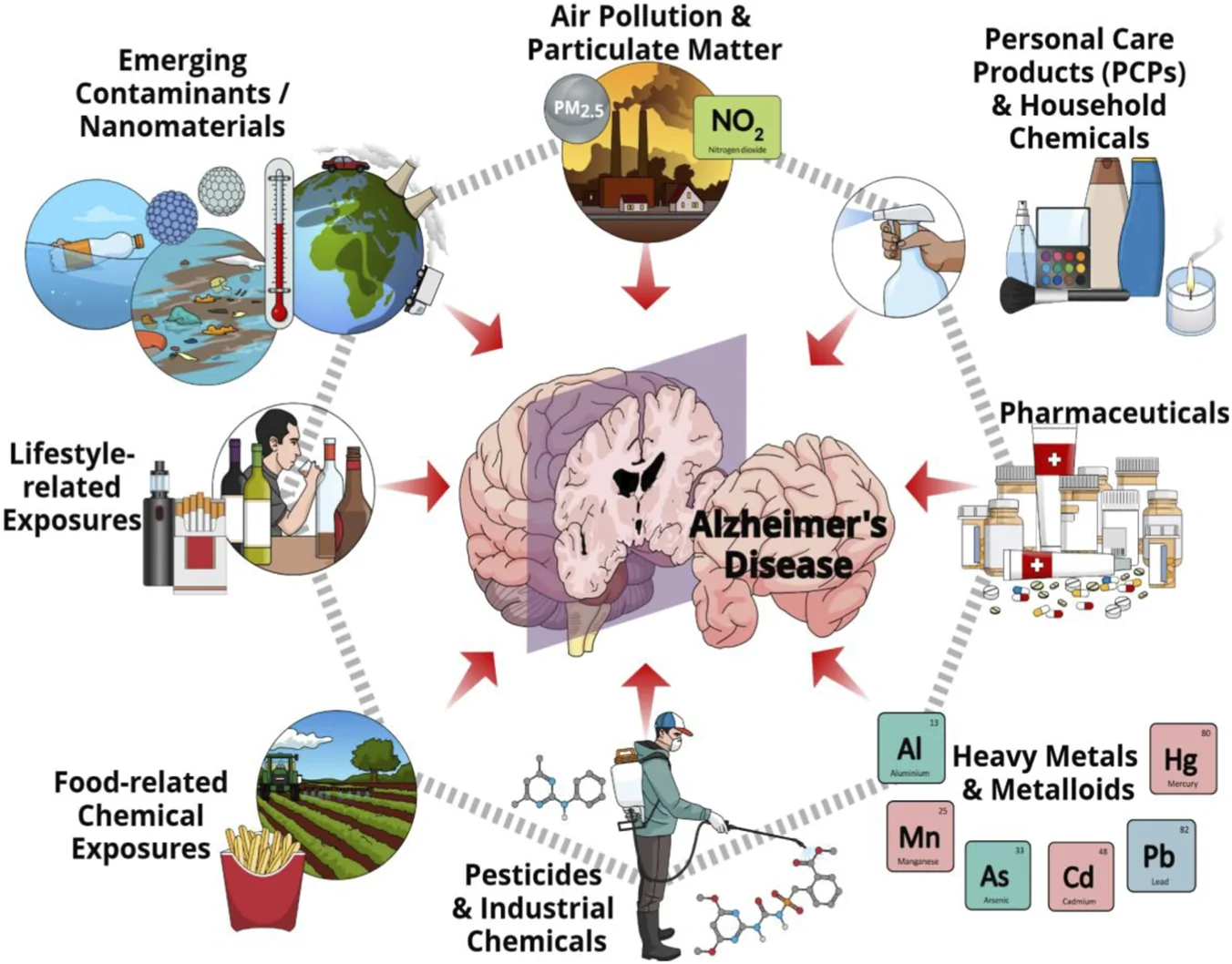
AD-related exposome.

In neurodegenerative diseases like AD, the “neural exposome” includes the combined toxic effects of medications, PCPs, and other environmental chemicals that may trigger or accelerate disease progression (Figure 3). While current data highlight the significant role of anticholinergic agents, air pollution, heavy metals, and pesticides, backed by multi-level evidence linking them to AD risk and pathology, certain other factors, such as food additives, microplastics, and nanomaterials, lifestyle choices like alcohol consumption, as well as ingredients of various PPCPs, require further investigation specifically related to AD (Table 1).

**TABLE 1:** An overview of the exposome in relation to dementia and the available reports on AD.

Main group	Specific examples	Key points/mechanisms in AD	Type of evidence	Selected references
Pharmaceuticals	Anticholinergics (e.g., diphenhydramine) Benzodiazepines Some anesthetics (e.g., isoflurane) Polypharmacy in the elderly	Anticholinergics: impaired cholinergic signaling (central in AD pathology: worsens cognitive reserve) ↑ Aβ production (anesthetics shown to increase BACE1 activity) Tau hyperphosphorylation (anesthesia-related) Sedatives linked to cognitive decline and increased dementia risk Chronic exposure may accelerate neurodegeneration via synaptic dysfunction	*In vitro* *In vivo* Human observational studies	Richardson et al. (2018), Coupland et al. (2019), Pieper et al. (2020), Zheng et al. (2021), Wu et al. (2023)
PCPs and household chemicals	Parabens Phthalates Bisphenols Triclosan (antimicrobial) Synthetic fragrances Disinfectants	Endocrine disruption → affects neuronal signaling ↑ Oxidative stress Microbiome disruption → gut-brain axis → neuroinflammation Triclosan and antimicrobials linked to immune dysregulation and neuroinflammation Chronic low-dose exposure may alter microbiome and brain–immune interactions Direct AD data remains limited	*In vitro* *In vivo* Limited human biomonitoring studies	Ren X. et al. (2023), Kiran et al. (2024), Du et al. (2025)
Air pollution and particulate matter	PM2.5, PM10 NO_2_ Diesel exhaust particles	↑ Aβ deposition Tau pathology acceleration Microglial activation→chronic neuroinflammation Blood-brain barrier (BBB) disruption ↑Oxidative stress Structural brain changes and cognitive decline	*In vivo* Epidemiology (large cohorts) Post-mortem	Calderón-Garcidueñas et al. (2008), Peters et al. (2019), Patten et al. (2021), Jahedi et al. (2025)
Heavy metals and metalloids	Lead (Pb) Mercury (Hg) Cadmium (Cd) Arsenic (As) Aluminum (Al)	Direct binding to Aβ → aggregation Tau hyperphosphorylation Epigenetic reprogramming (e.g., Pb exposure early-life → later Aβ dysregulation) Mitochondrial dysfunction ROS → neuronal death Cross BBB and accumulate in central nervous system	*In vitro* (protein aggregation assays) *In vivo* Epidemiology Post-mortem	O’Bryant et al. (2011), Huat et al. (2019), Islam et al. (2022), Althobaiti (2025)
Pesticides and industrial chemicals	Organophosphates Organochlorines (e.g., DDT) Solvents PCBs	Long-term exposure linked to increased AD risk Acetylcholinesterase inhibition (organophosphates) → cholinergic stress Mitochondrial dysfunction and oxidative stress ↑ Aβ production and impaired clearance Microglial activation Endocrine disruption → tau effects	*In vitro* *In vivo* Occupational epidemiology Biomarker cohort data	Yan et al. (2016), Bartholomew et al. (2024), Ruiz-González et al. (2024)
Food-related chemical exposures	Contaminants (heavy metals and metalloids, pesticide residues) Advanced glycation end products (AGEs) Ultra-processed food additives	Diet is a major exposome component Contaminants contribute to inflammation and oxidative stress May influence gut–brain axis and amyloid metabolism AGEs → bind RAGE receptor → ↑ Aβ transport into brain Gut microbiome alteration → systemic inflammation → microglial activation	*In vitro* (AGE–RAGE signaling) *In vivo* Human dietary cohort studies	O’Bryant et al. (2011), Li et al. (2021), Zhu G. et al. (2023), Ahmed et al. (2025), Boccardi et al. (2025)
Lifestyle-related chemical exposures	Tobacco smoke Alcohol (particularly alcohol use disorders)	Smoking and alcohol → oxidative stress, vascular damage, neuroinflammation Synergistic effects with environmental toxins ROS → oxidative stress BBB disruption Microglial activation ↑Aβ aggregation (smoke-related metals/particles) Tau phosphorylation (alcohol use disorder)	*In vivo* Human longitudinal studies Post-mortem	Almeida et al. (2008), Monnig (2024), Chang et al. (2025)
Emerging contaminants/nanomaterials (nanoparticles, microplastics)	Nanoparticles (metal-based) Micro-/nano-plastics (emerging concern)	Cross biological barriers, including the BBB Potential to induce neurotoxicity and inflammation Increasing concern in exposome research: Potential co-exposure effects remain poorly understood Microglial activation Protein misfolding (potential Aβ/tau interactions) Oxidative stress	*In vitro* *In vivo* (limited) Very limited human data	Rahman et al. (2020), Kopatz et al. (2023), Zhang X. et al. (2024), Dai et al. (2025)

## 4 Pharmaceuticals

As summarized in Tables 1,2, the pharmaceutical component of the exposome includes both prescription drugs and over-the-counter medications that may affect neurodegeneration risk (La Cognata et al., 2021; Gao et al., 2022; Iturria-Medina et al., 2022; Khanna and Jones, 2023).

**TABLE 2:** Overview of the dementia risk in relation to the most commonly evaluated pharmaceuticals.

Drug class	Mechanism	Association with AD	Exposure assessment	Evidence level	Ref.
Central anticholinergic agents	Central muscarinic receptor antagonism → reduced acetyl-choline signaling → impaired hippocampal synaptic plasticity and memory dysfunction	Dose-dependent increase in dementia incidence; some subclasses (urological, anti-Parkinson, antidepressant) consistently harmful; some CV/GI agents possibly neutral or protective; polypharmacy of anticholinergics shows “stair step” increase in dementia and mortality	High cumulative anticholinergic burden and longer exposure → higher risk; sex effects not well-characterized in human data; long-term use	Moderate/high-level (meta-analyses, large case-control, prospective cohort evidence; observational)	Gray et al. (2015), Richardson et al. (2018), Coupland et al. (2019), Zheng et al. (2021), Poonawalla et al. (2023)
Benzodiazepines	GABA-_A_ receptor modulation → enhanced inhibitory neurotransmission → impaired memory consolidation	Association between long-term use and increased dementia risk	Duration of use (>3 months), cumulative exposure	Moderate observational evidence	Billioti de Gage et al. (2014), Zhong et al. (2015), Cortes-Flores et al. (2024), Underwood et al. (2025)
Antipsychotic agents	Dopamine D_2_ receptor antagonism + cerebrovascular adverse effects	Increased mortality and clinical deterioration in dementia patients	Polypharmacy, frailty, vascular comorbidity	Clinical observational + regulatory evidence	Kales et al. (2012), Cortes-Flores et al. (2024), Underwood et al. (2025)
Proton pump inhibitors (PPIs)	Vitamin B_12_ deficiency and lysosomal dysfunction (hypothesized)	Inconsistent findings; possible increased risk in some cohorts	Long-term use	Low/moderate, conflicting cohort evidence	Haenisch et al. (2015), Gomm et al. (2016)
Antidiabetic agents (metformin, SGLT-2i, GLP-1 RA; DPP-4i)	Modulation of insulin signaling and neuronal metabolism; potential neuro-protection via various mechanisms	Conflicting results; confounding by earlier disease stage and comorbidity burden is likely	Diabetes duration, dosage	Moderate heterogeneous evidence	Imfeld et al. (2012), Ng et al. (2014), Nemeh et al. (2025), Sun et al. (2025), Zhang et al. (2025)
Acetylcholine esterase inhibitors	Inhibition of Ach degradation → increased synaptic cholinergic transmission	Symptomatic cognitive improvement (no disease modification)	Disease stage	High-level RCT evidence	Birks and Harvey (2006)
Disease-modifying drugs for AD (lecanemab, donanemab)	Reduce Aβ burden, potentially targeting pTau, and other mechanisms	Reduction in amyloid levels vs. placebo in early AD, deceleration of cognitive/functional decline; long-term safety profile and effects on incidence are uncertain	Given during early symptomatic stages; exposure characterized by trial dosing	Moderate–high for amyloid lowering; modest clinical effect; uncertain population-level prevention	Peng et al. (2023), Popov et al. (2024), Zimmer et al. (2026)
HMG-CoA reductase inhibitors (statins)	Cholesterol reduction → altered amyloid precursor protein processing + anti-inflammatory effects	Some cohort studies show reduced AD	Long-term exposure, early initiation	Moderate epidemiological evidence	Jick et al. (2000), Haag et al. (2009)
NSAID	COX inhibition → reduced neuroinflammation	Protective signal in observational studies; not confirmed in RCTs	Timing of exposure (preclinical phase)	Moderate evidence, some inconsistency	Aisen et al. (2003), Launer (2003)
Antihypertensive agents (ACE inhibitors/ARBs)	Improved cerebral perfusion and vascular protection	Consistent association with reduced risk of all-cause dementia (treating hypertension vs. not), class differences	Duration of blood pressure control	Moderate/high-level randomized + cohort evidence	Williamson et al. (2019), Sible and Nation (2023), Singh et al. (2026)
Antibiotics	Conflicting results (alteration to gut microbiota → Aβ deposition, tau pathology, microglial activation, and neuroinflammation vs. ineffective vs. beneficial effects)	Conflicting results	Cumulative burden Long-term use	Low to moderate heterogeneous evidence	Portero-Tresserra et al. (2018), Howard et al. (2020), Ye et al. (2024)

### 4.1 Polypharmacy

Given that the majority of adults diagnosed with AD are adults aged 65 years or older, who frequently have comorbid conditions such as hypertension, diabetes, cardiovascular disorders, osteoporosis, prostate-related conditions, and gastrointestinal diseases (Sharma et al., 2024), AD patients are more prone to polypharmacy compared to other population groups, leading to an increased risk of adverse drug reactions and drug-drug interactions. A systematic review and meta-analysis found that cognitive impairment risk increases in older adults taking ≥5 medications, rising further with ≥10 medications, though causality remains to be confirmed (Yu X. et al., 2024).

Moreover, a study reported that polypharmacy is more common among dementia patients compared to other elderly people, not only for the treatment of dementia symptoms but also targeting cardiovascular, urological, and gastrointestinal systems (Growdon et al., 2021). To date, several medications are implicated in dementia, including anticholinergics, proton pump inhibitors (PPIs), benzodiazepines, and analgesics (Gray et al., 2015; Grossi et al., 2019; Chen et al., 2020; Joyce et al., 2022; Pourhadi et al., 2024). Including these drugs in polypharmacy may exacerbate the general condition of AD patients and increase AD risk in elderly non-dementia patients. A study conducted with 1,420 participants whose mean age was 71.2 reported that analgesics were prescribed to 84% of them during 1 year, making them one of the most common drugs involved in polypharmacy in the elderly. Among them, NSAIDs were the most common (77%), followed by paracetamol (41%), opioids (32%), gabapentinoids (17%), and tricyclic antidepressants (7%) (Marttinen et al., 2021). A recent study found that regular opioid usage is associated with a 20% higher risk of dementia, while using strong opioids is linked to 72% higher risk of dementia compared to non-users. Interestingly, despite regular use of strong opioids causing a decrease in hippocampal volume, no link was found between opioids and the increase in AD risk (Lin T. et al., 2025). Long-term exposure to anticholinergic drugs has been associated with an increased risk of incident dementia, with some evidence suggesting a dose-response relationship (Coupland et al., 2019; Joung et al., 2019; Zheng et al., 2021). Patients with established dementia experience higher rates of polypharmacy, including anticholinergic drugs, and are more likely than those without dementia to receive these medications during outpatient care (Growdon et al., 2021). This prescribing pattern is clinically concerning because anticholinergic drugs may reduce the effectiveness of cholinesterase inhibitors in AD. Given the modest efficacy of cholinesterase inhibitors and the limited pharmacological options for AD, minimizing unnecessary anticholinergic exposure remains important. Available therapies include adjunctive memantine, typically used in combination with cholinesterase inhibitors, and anti-Aβ monoclonal antibodies, whose use remains limited by safety concerns and high costs (Qiao et al., 2024; Yang, 2025).

A critical consideration when evaluating the association between anticholinergic drugs and AD risk is to consider that there are several drug classes with anticholinergic properties, such as analgesics, antiemetics, antidepressants, antiepileptics, cardiovascular, antipsychotic, respiratory, gastrointestinal, urological, and anti-Parkinson drugs, since they all may exhibit different effects on AD risk (Richardson et al., 2018; Coupland et al., 2019; Liu et al., 2020; Zheng et al., 2021). For instance, especially antidepressants, anti-Parkinson and urological drugs, are reportedly increasing AD risk significantly, while anticholinergic gastrointestinal and cardiovascular drugs are linked to a minor decrease in AD risk (Gray et al., 2015; Chatterjee et al., 2016; Richardson et al., 2018; Coupland et al., 2019; Liu et al., 2020; Zheng et al., 2021). It should be noted that anticholinergic drugs span several drug classes, and risk analysis for each one of them should be accordingly. Since anticholinergics have various indications, one or multiple drug classes in this category might be part of polypharmacy in AD patients. Avoiding or promoting anticholinergic use in this population based solely on the classification may be misleading; thorough research into each category is necessary before reaching a final decision.

### 4.2 Cumulative burden

In AD patients, altered drug metabolism and elimination resulting from age-related changes in hepatic and renal function, long-term polypharmacy, and the use of medications aimed at managing disease symptoms collectively lead to a cumulative drug burden. Conducting research in this area is highly challenging and costly due to the need for long-term follow-up and pharmacogenetic profiling, the potential for drug interactions among medications used by patients in this age group, reverse causality issues, and uncertainty regarding adherence to prescribed medications. Research has generally focused on the relationship between anticholinergic burden and the risk of AD. A recent study reported that the cumulative exposure to strong anticholinergics is associated with dementia, especially among men and patients diagnosed at a younger age. The association between dementia risk and drug burden was dependent on cumulative dose, anticholinergic potency, and drug class. Especially urinary antispasmodics, psychotropic medications, and antihistamine burden among strong anticholinergic classes elevated dementia risk in a dose-dependent manner, while musculoskeletal and gastrointestinal system drugs showed no association. Compared to AD, the risk of vascular dementia and Lewy body dementia increased more due to anticholinergics (Zhu N. et al., 2025). Another study demonstrated that exposure to one or more anticholinergic drugs was linked to increased risks of dementia/AD-1.6, 2.1, 2.6, and 2.6 times higher for one, two, three, and four or more drugs, respectively, and elevated mortality risk, 1.4, 2.6, 3.8, and 3.4 times, respectively, vs. no exposure (Poonawalla et al., 2023).

A recent study Decaix et al. (2026) reported that 21% of AD patients had a high anticholinergic burden, while polypharmacy was observed in 42.5%. At the end of 7.3 follow-up years, 51% of patients had deceased, and it was found that polypharmacy was common among these patients, while anticholinergic burden was significantly more heightened. Although polypharmacy has been found to increase mortality risk in older adults by %28 in a meta-analysis, Li et al. (2022) anticholinergic burden and potentially inappropriate medications related to cognition linked to mortality more than polypharmacy alone (Li et al., 2022; Decaix et al., 2026).

Overall, the use of anticholinergic medications in patients with AD should be carefully weighed in terms of the benefit–to–risk balance and closely monitored in clinical practice. In risk groups such as older adults, avoiding unnecessary use of anticholinergic medications may be beneficial for AD prevention. However, the current literature on the relationship between cumulative medication burden and AD has mainly focused on anticholinergic drugs, which represent an important limitation. More comprehensive studies are needed to evaluate the association between drug classes and AD, taking into account factors like dosage, duration of use, comorbidities, and concomitant medication use. Expanding current literature may enhance our understanding of medication-related risks and support effective strategies for both disease prevention and prognosis.

## 5 PCPs and household chemicals

PCPs and household chemicals represent underappreciated contributors to the cumulative exposome burden. Long-term exposure to environmental contaminants has been linked to AD via mechanisms such as oxidative stress, neuroinflammation, protein dysregulation, epigenetic alterations, immune dysregulation, and mitochondrial dysfunction (Huat et al., 2019; Ahmed et al., 2025). Systems-level analyses emphasize that bisphenols, heavy metals, and persistent organic pollutants exhibit strong mechanistic connectivity to these pathways (Hong, 2025). Beyond established toxicants like air pollutants, heavy metals, and pesticides, EDCs may contribute to AD risk by impairing pancreatic function, insulin signaling, lipid metabolism, and brain insulin sensitivity, thereby linking exposure to metabolic dysfunction and cognitive decline (Johri et al., 2024). Given the prevalence of chronic low-level exposures, PCPs and household chemicals should be prioritized in prevention-oriented research and risk assessment. Future studies must account for complex mixture exposures, integrating co-exposures with genetic susceptibility, lifestyle, and environmental factors to better characterize mixture toxicity and its translational implications (Ducroq et al., 2023; Johri et al., 2024; Wang et al., 2025).

### 5.1 Phthalates

Phthalates are ubiquitous EDCs also found in PCPs, medical devices, food-contact materials, toys, and other plastic consumer goods, resulting in chronic, low-level exposure (Luís et al., 2021; Eales et al., 2022). Not covalently bound to polymer matrices, they readily leach into the environment, and their ability to cross the placental barrier makes prenatal and early-life exposure a critical concern for developmental toxicity (Qian et al., 2020; Lucaccioni et al., 2021; Tran et al., 2023; Johri et al., 2024).

Epidemiological evidence links phthalate exposure to adverse neurodevelopmental outcomes (Lucaccioni et al., 2021; Eales et al., 2022). Although direct epidemiological evidence linking phthalates to AD is limited, a recent meta-analysis reported a positive association between EDC exposure and increased AD risk, supporting the hypothesis that endocrine disruption may contribute to neurodegeneration and highlighting the need for further cohort, *in vivo*, and *in vitro* studies to elucidate mechanisms (Wang et al., 2025). Experimental studies provide mechanistic support for these associations. Di (2-ethylhexyl) phthalate (DEHP) and related compounds induce oxidative stress, mitochondrial dysfunction, neuroinflammation, BBB disruption, impaired neuronal differentiation, apoptotic neuronal injury, and, in some experimental models, sex-specific neurotoxic effects (Benjamin et al., 2017; Ducroq et al., 2023; Liu et al., 2023; Ren W. et al., 2023; van Melis et al., 2025). Recent network toxicology and single-cell transcriptomic analyses further implicate AD-related molecular pathways in phthalate neurotoxicity (Li et al., 2026). Prenatal DEHP exposure has also been reported to activate JAK2/STAT3 signaling in offspring, linked to AD and a potential therapeutic target. Additionally, maternal DEHP increases pro-inflammatory factors damaging the central nervous system and associates with microglial activation in offspring (Wei et al., 2024; Chen et al., 2026). Proposed epigenetic mechanisms link early-life exposures to later neurological vulnerability, involving placental DNA hypomethylation and altered methylation of genes associated with neurological disorders (Tran et al., 2023).

### 5.2 Parabens

Another class of EDCs, parabens, found in PCPs and food packaging, can cross the BBB and the placenta, raising concerns regarding developmental neurotoxicity (Denuzière and Ghersi-Egea, 2022). Recent evidence indicates that prenatal and postnatal exposure to butylparaben reduces cell viability and neural differentiation, and induces sex-specific behavioral changes in offspring mice. Reportedly, male offspring exposed to butylparaben exhibited memory impairment and social dysfunction, whereas female offspring showed increased compulsive behavior and subtle spatial memory impairment. These sex-specific behavioral outcomes are attributed to the estrogenic and androgenic activities of butylparaben. Mechanistically, exposure to butylparaben was associated with a dose-dependent increase in acetylcholinesterase gene expression and NMDAR expression in female mice (Kim et al., 2025). Both of these alterations are among the most well-known pathophysiological mechanisms in AD and represent the limited available therapeutic targets commonly used. Although existing evidence largely stems from experimental models, these findings bolster the biological plausibility that sustained paraben exposure could contribute to enduring neurodevelopmental changes and potentially elevate the risk of subsequent neurodegenerative conditions, thereby underscoring the need for comprehensive epidemiological research.

### 5.3 Bisphenols

Bisphenols, representing another class of EDCs, are widely used in polycarbonate plastics, epoxy resins, and consumer products, and growing evidence suggests that exposure may contribute to cognitive dysfunction and neurodegeneration, although evidence varies across individual analogs and experimental models (Costa and Cairrao, 2024; Xu et al., 2024). Bisphenol A (BPA), the best-characterized analog, impairs learning and memory, disrupts synaptic plasticity and neurotransmitter regulation, and induces oxidative stress, neuroinflammation, apoptosis, and neuronal injury (Suresh et al., 2022; Costa and Cairrao, 2024). Experimental data demonstrate AD-relevant alterations, including increased Aβ accumulation, tau hyperphosphorylation, cholinergic dysfunction, and impaired insulin signaling, providing mechanistic support for BPA-associated neurodegeneration (Wang et al., 2017; Flores et al., 2022). Network toxicology approaches identify convergent pathways linking BPA and phthalate neurotoxicity, including oxidative stress, mitochondrial dysfunction, metabolic dysregulation, and inflammatory signaling, overlapping with AD-related networks (Xu S. et al., 2025; Zhang et al., 2026).

Developmental exposure may represent a particularly critical window, as prenatal BPA exposure has been associated with persistent cognitive and neurodevelopmental alterations in offspring (Suresh et al., 2022). Importantly, evidence indicates that BPA substitutes may also possess biologically relevant neurotoxicity. In experimental models, bisphenol F (BPF) and bisphenol S (BPS) have been shown to impair neural stem-cell function, hippocampal neurogenesis, learning, and memory (Tiwari et al., 2024; Cantua and Mulligan, 2025); specifically, BPF exposure has been associated with impaired cognitive performance in zebrafish through altered neural cellular responses (Mu et al., 2022).

Although direct human evidence linking bisphenols to dementia remains limited, emerging studies associate certain bisphenol analogs with Aβ positivity, cognitive impairment, and heightened risk indicators in older populations, though causality remains unresolved (Canovas et al., 2025; Deng et al., 2025). Bisphenols are considered plausible environmental contributors to AD-related neurodegenerative processes; however, prospective human studies that incorporate environmentally relevant exposures, chemical mixtures, and exposome-informed approaches are necessary to elucidate specific risks and causal relevance to dementia (Costa and Cairrao, 2024; Xu et al., 2024).

Collectively, EDCs—including phthalates, parabens, and bisphenols—are associated with pathogenic pathways implicated in AD, such as oxidative stress, mitochondrial dysfunction, neuroinflammation, epigenetic dysregulation, and impaired proteostasis. Although the strength of evidence varies across chemical classes and experimental models, shared mechanisms support biological plausibility of EDCs contributing to AD-related neurodegeneration. Establishing causality requires prospective human studies incorporating environmentally relevant exposure assessments, chemical-mixture analyses, and exposome-based multi-omic approaches to clarify individual and combined impacts on disease risk.

### 5.4 Per- and polyfluoroalkyl substances (PFAS)

PFAS are persistent environmental contaminants detected in diverse consumer products, including cosmetics, food-contact materials, textiles, and stain-resistant coatings. Their widespread exposure has raised concerns about neurological effects, with experimental and epidemiological evidence implicating oxidative stress, neuroinflammation, mitochondrial dysfunction, endocrine disruption, and altered lipid metabolism in neurodevelopmental and cognitive outcomes (Song et al., 2026). Recent findings in cerebral organoid models indicate that common PFAS mixtures can induce AD-like neuropathological signatures (Lu et al., 2024). Although human evidence linking PFAS exposure to cognitive impairment and dementia remains limited, their biological persistence and overlap with established AD-related pathways underscore the need for detailed investigation within an exposome-informed framework (Gardener et al., 2025). Future longitudinal studies integrating mixture-based exposure assessment, biomonitoring, and multi-omic profiling are needed to clarify the potential contribution of PFAS and other persistent contaminants to AD risk.

### 5.5 Heavy metals and metalloids

Heavy metals and metalloids are of particular interest because they can disrupt metal homeostasis, promote oxidative stress, and influence key neurodegenerative pathways. The metal hypothesis of AD proposes that age-related impairment of metal regulation contributes to disease progression through mechanisms involving Aβ aggregation, tau dysregulation, oxidative damage, mitochondrial dysfunction, and neuroinflammation. Chronic exposure to toxic metals, including lead (Pb), cadmium (Cd), and mercury (Hg), as well as dysregulation of essential metals such as iron (Fe), copper (Cu), and manganese (Mn), has been associated with altered neural metal balance and neurotoxic effects. These findings highlight metal-related environmental exposures as potential modifiable contributors to AD pathogenesis (Doroszkiewicz et al., 2023). Studies indicate that these metals impair mitochondrial function and increase ROS generation. The resulting oxidative stress may promote Aβ aggregation, plaque formation, and synaptic dysfunction (Babić Leko et al., 2022; Islam et al., 2022). For arsenic (As), Mn, Pb, and Cd, these metals share common mechanisms, including oxidative stress, mitochondrial dysfunction, protein aggregation, neuroinflammation, impaired autophagy, and tau hyperphosphorylation. Additionally, Pb disrupts the BBB and epigenetically alters AD genes; Cd induces neuronal aging via the p53/p21/Rb pathway; As impairs nitric oxide (NO) signaling and cortical/synaptic function; and Mn causes glutamate excitotoxicity and dopaminergic neuron damage (Wang L. et al., 2020; Ahmed et al., 2025; Antinori et al., 2025). This process also contributes to disease progression by disrupting metal balance and increasing neuroinflammation (Babić Leko et al., 2022). The most significant biological effect of metal exposure is disruption of cellular redox balance. Metal ions participate in redox reactions, leading to excessive free radical generation and thereby causing oxidative stress by overwhelming antioxidant defense mechanisms. In particular, metals such as excess Fe produce hydroxyl radicals via the Fenton reaction and increase oxidative stress. This significantly contributes to neuronal dysfunction and cell death through cellular lipid peroxidation, protein damage, and DNA damage. All these damages contribute to the progression of AD pathology by leading to neuronal dysfunction and, over time, cell death (Islam et al., 2022; Pyatha et al., 2022). A 2025 meta-analysis showed that exposure to environmental aluminum may preferentially accumulate in brain cells that produce neurofibrillary tangles, and reported a statistical association, suggesting that aluminum may increase the risk of the disease (Soleimani et al., 2025).

### 5.6 Pesticides

Pesticide use is now regarded as unavoidable, with an estimated one-third of agricultural products involved (Pszczolińska et al., 2024). A study revealed that prenatal chlorpyrifos exposure leads to decreased white matter volume and increased thickness in frontal, temporal, and postero-inferior cortices, along with sustained reductions in brain metabolism and cerebral blood flow. It also correlates with diminished fine motor and motor programming skills, although brain metrics did not significantly mediate these relationships (Peterson et al., 2025). Studies conducted on individuals exposed to pesticides during the prenatal period are significant for determining the effects of pesticides on neurodevelopmental processes, and the extent to which these effects lead to alterations in brain function, brain structure, and cognitive performance. Longitudinal studies involving children with prenatal pesticide exposure, followed into later stages of life, may be useful in determining the extent and strength of the association between cumulative pesticide exposure and AD, as well as other neurological disorders in older age. A meta-analysis reported a highly significant link between AD and β-HCH, dieldrin, and organochlorine pesticides, particularly DDE (Zayani et al., 2026). Although these pesticides are banned today, several studies report their occurrence worldwide due to environmental residues and illegal use, highlighting the need for various strategies to be implemented to avoid their harmful effects (Wang H. et al., 2020; Anaduaka et al., 2023; Alaoui et al., 2024; Sapkota et al., 2025). Primarily, comprehensive, systematic global studies are essential for detecting pesticide residues in the environment and determining their prevalence and distribution. Additionally, conducting cohort studies in populations exposed to banned pesticides would be beneficial for strengthening causal inference, assessing the dose-response relationship, and evaluating the effects of pesticide residues and environmental interactions on human health. Strengthened surveillance and enforcement measures are required to prevent or strictly control the inappropriate use and trade of banned or restricted pesticides, and more systematic pesticide monitoring of agricultural products may be beneficial, particularly for those cultivating in agricultural regions where previously reported use of such pesticides.

### 5.7 Solvents

Organic solvents constitute a common class of occupational and household chemicals with well-established neurotoxic potential. While epidemiological studies and experimental data indicate solvents as potential risk factors for neurodegenerative diseases, direct evidence specifically linking solvent exposure to AD remains scarce (Kulcsárová et al., 2025). Current literature shows stronger associations with other neurodegenerative conditions, and the correlation with AD is yet to be definitively explored.

## 6 An overview of synergistic mechanisms and primary organelle targets

**FIGURE 4:**
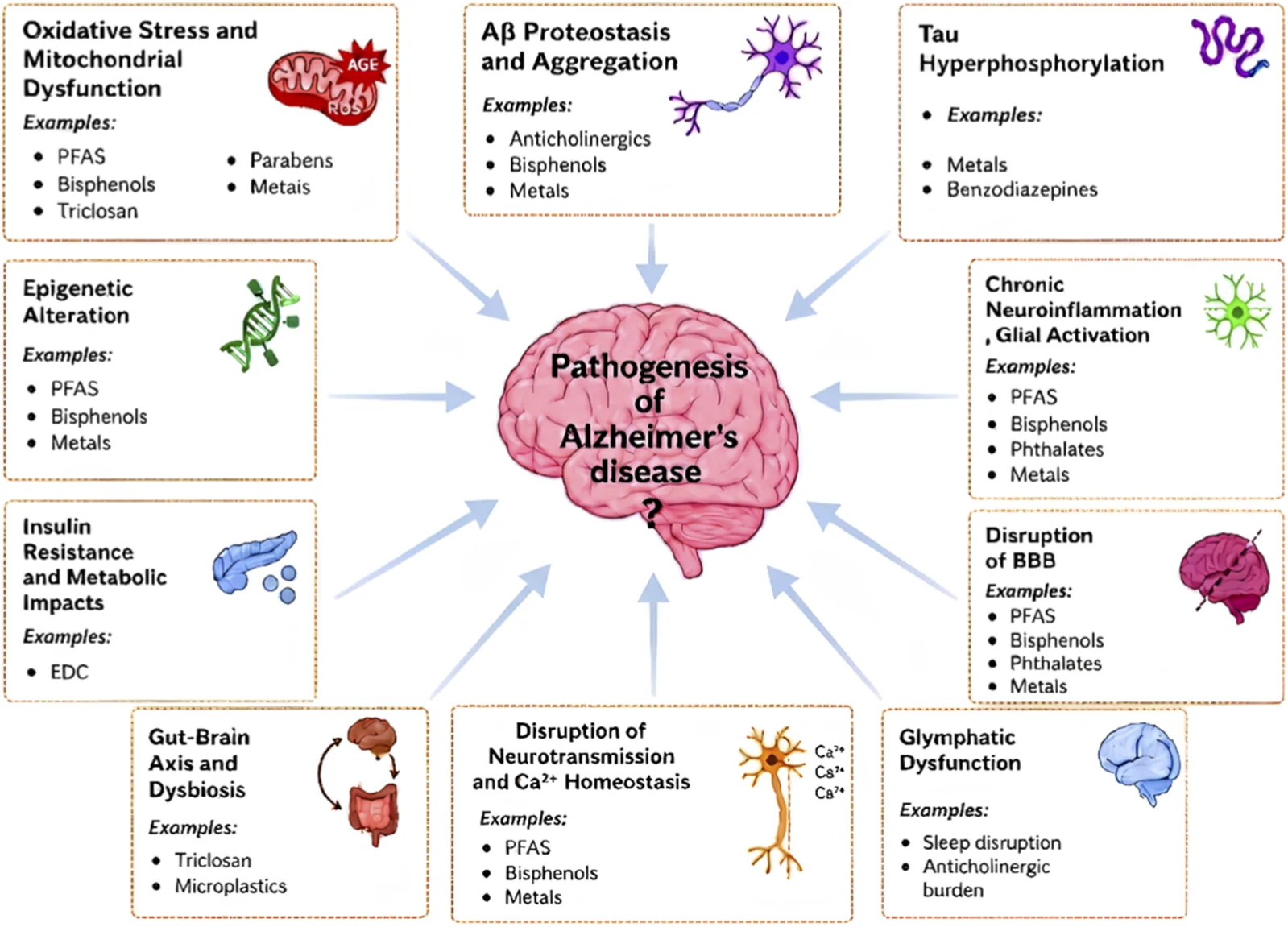
An overview of AD pathological mechanisms and selected examples.

Environmental and chemical exposures seldom occur in isolation; rather, their interactions can synergistically induce neurotoxic effects, such as neuroinflammation, mitochondrial dysfunction, and oxidative stress (Figure 4).

For example, exposure to phenols, parabens, and phthalates may adversely impact cognition in older adults, and urinary measurements of these chemicals are also associated with cognitive decline in populations with low-level exposure. Combined exposure correlates with lower cognitive test scores, with MECP exposure exerting a predominant influence in males (Du et al., 2025). Exposure to metal mixtures such as As, Cd, and Pb in rats induces oxidative stress–dependent neuroinflammation, upregulates APP and BACE, increases Aβ levels, and results in cognitive impairment that exceeds the effects observed with individual metals, indicating a synergistic interaction, with the tertiary mixture causing elevated Aβ levels and consequent cognitive deficits in young rats. Among individual metals, Pb elicited the greatest Aβ induction, while As and Cd had minimal effects despite increasing APP levels due to reduced BACE and presenilin induction; when combined, these metals synergistically enhanced the effect, mainly driven by As (Ashok et al., 2015).

In light of organelle-level dysfunction in AD, including mitochondria, lysosomes, endoplasmic reticulum, and others (Gao et al., 2025), research in the neural exposome is expected to increasingly concentrate on these emerging targets for xenobiotics. Mitochondrial homeostasis plays a vital role in sustaining cellular energy metabolism, calcium signaling, and apoptosis, and its dysfunction has been linked to AD pathogenesis via impaired electron transport, increased ROS, and oxidative stress. Mitochondria are increasingly acknowledged as notable biomarkers for early diagnosis and disease monitoring, as well as promising therapeutic targets for AD (D’Alessandro et al., 2025). Exposure to phthalates and bisphenols has been linked to alterations in mtDNA methylation, some of which are connected to increased oxidative stress through elevated ROS production, disruptions in redox balance, and the generation of extracellular superoxide (Reddam et al., 2022). A study on a highly effective benzodiazepine, alprazolam, has shown that repeated use leads to diminished hippocampus-dependent memory consolidation rather than impairing memory acquisition, and this adverse effect, caused by mitochondrial dysfunction, can be prevented (Zhu S. et al., 2023). Other organelles, such as the Golgi apparatus (Fourriere and Gleeson, 2025), endoplasmic reticulum (Ajoolabady et al., 2022), lysosomes (Yuan et al., 2023), and membrane contact sites (Girolimetti et al., 2025) have also been implicated.

## 7 Key omics layers in exposome research

**FIGURE 5:**
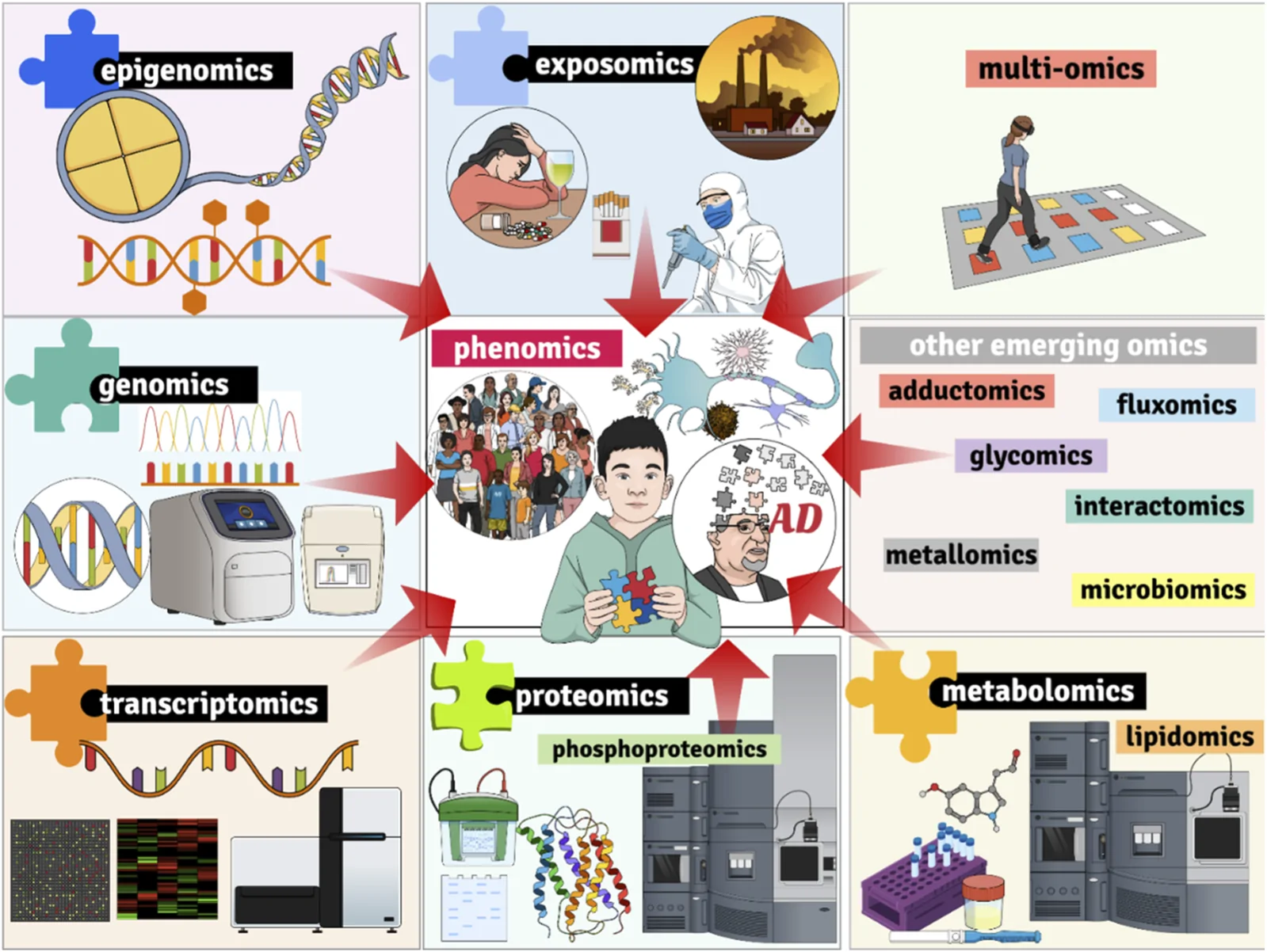
Omics in exposome research.

**FIGURE 6:**
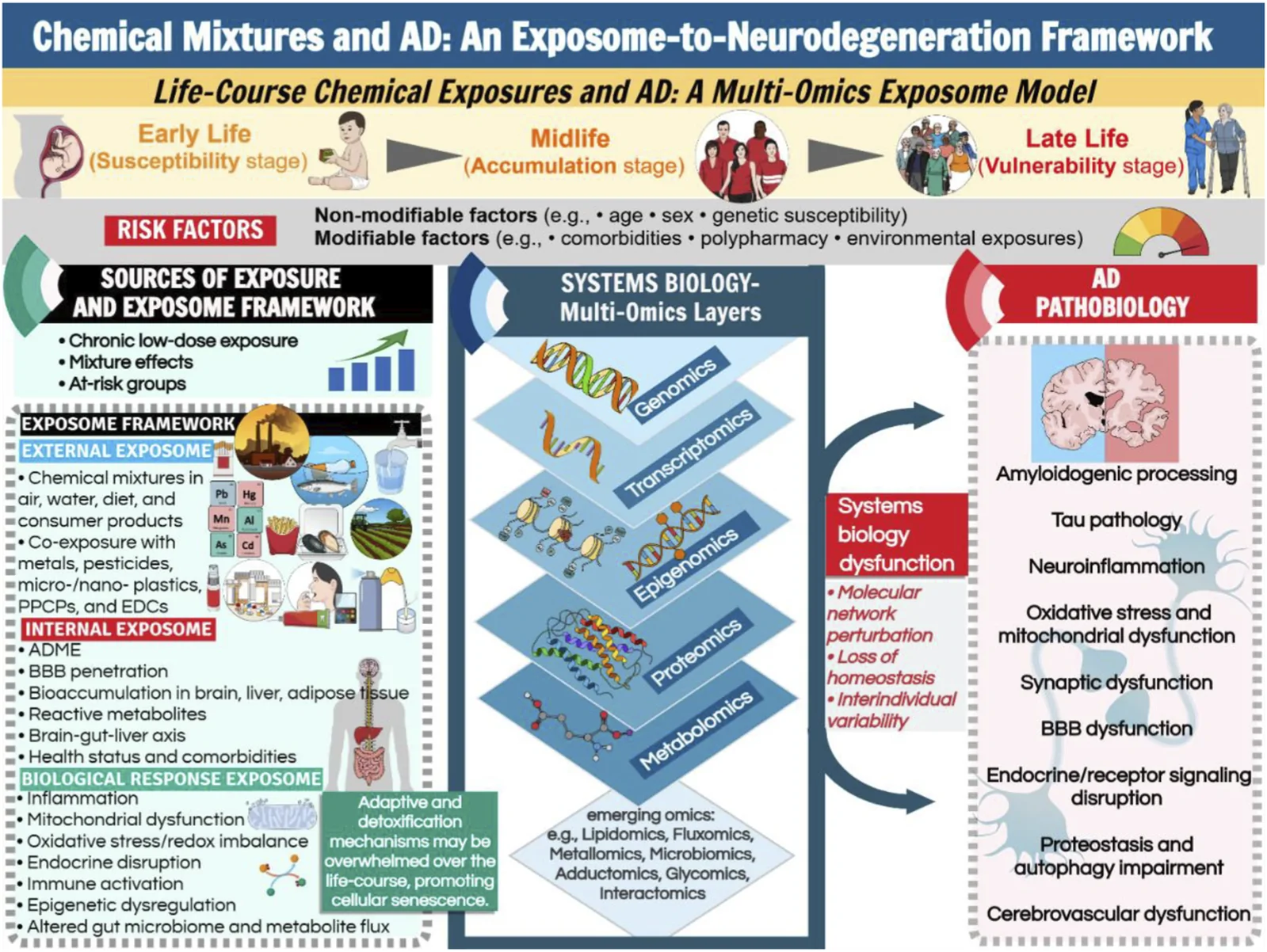
An overview linking environmental exposures, exposome components, omics data layers, and molecular pathways related to AD.

The suffix -ome denotes entire sets of molecular components—such as genome (DNA), transcriptome (RNA), proteome (proteins), and metabolome (metabolites)—each studied within their respective -omics fields, genomics, transcriptomics, proteomics, and metabolomics, which rely on high-throughput methods for comprehensive analysis. While these four core omics are the most developed and applied, they are not the only ones. Others, such as epigenomics—which examines transient chemical modifications of DNA—and lipidomics—focused on the lipidic subpopulation of metabolites—are emerging fields that elucidate cellular function, necessitating complex interplay among different omics levels (Poinsignon et al., 2023). While individual “omics’ offer snapshots of molecular states, their integrated analysis—encompassing genomics, transcriptomics, proteomics, and metabolomics, as well as others—is crucial for understanding how exposures or the exposome influence biological systems and contribute to AD (Figure 5, Table 3). By integrating interactions across genetic, epigenetic, proteomic, metabolomic, and environmental factors, systems-level approaches facilitate formulation of mechanistically grounded, testable hypotheses regarding AD initiation, progression, and potential therapeutic targets, thereby advancing towards predictive models and biomarker-driven precision medicine tailored to individual molecular profiles (Bice et al., 2026). A systematic review of recent omic studies identified proteomics as the most frequently studied layer, often integrated with transcriptomics in AD multi-omics research (Dong and Zhong, 2025). The key issues were summarized as: Proteomics offers a distinctive perspective: while genomics and transcriptomics identify upstream risk factors and regulatory alterations, proteomics directly assesses the effectors of cellular dysfunction. Notable discrepancies between transcripts and proteins highlight layers of translational regulation and protein turnover, underscoring the importance of integration of proteomics into multi-omics approaches to link genetic variants, pathways, and phenotypes in AD. Including proteomics in well-designed multi-omics studies offers vital insights into AD biology, especially as emerging technologies, larger cohorts, and growing disease burden call for mechanism-based strategies (Dong and Zhong, 2025). Figure 6 illustrates the complex interplay between environmental exposures, exposome components, omics layers, and molecular pathways associated with AD, underscoring the external, internal, and biological response exposome, as well as the detoxification systems, which can become increasingly overwhelmed over the lifespan due to cumulative low-dose exposures, chemical mixture effects, and age-related metabolic decline.

**TABLE 3:** Examples of key omics layers in exposome research.

Toxicants	Omics layers/approaches	Key mechanisms and findings	Selected reference
MEP	Transcriptomics, proteomics, molecular docking analysis	A causal relationship was found between MEP and AD. Six core genes, namely *EGFR, MAPK3, MMP9, TP53, ESR1*, and *PTGS2*, are involved in the pathogenesis of AD. *EGFR* expression was notably increased in the entorhinal cortex, hippocampus, and temporal cortex. Conversely, PTGS2 was specifically downregulated in the temporal cortex; the other core genes (TP53, ESR1, MAPK3, MMP9) showed no significant differential expression across brain regions Functional enrichment highlighted neuronal signaling, inflammation, and metabolic processes	Gong et al. (2025)
DEHP	Transcriptomics	Exposure of *Caenorhabditis elegans* AD models (strains CL4176 and CL2006) to DEHP during early developmental stages resulted in heightened Aβ toxicity Early-life and chronic DEHP exposure significantly increased intracellular ROS and Aβ levels, potentially linked to the autophagy–lysosomal degradation pathway in aged *C. elegans* CL2006 strains DEHP-induced Aβ toxicity occurs independently of the transcription factors DAF-16 and SKN-1, whereas early-life and chronic DEHP exposure markedly elevate lysosome-related organelle accumulation and increase bec-1 mRNA levels in aged CL2006	Yen et al. (2021)
BPA	Transcriptomics, interactomics, targeted gene expression analysis (quantitative RT-PCR), protein expression analysis (Western blotting)	Maternal BPA exposure may elevate offspring’s risk by disrupting genes linked to AD neuropathology and inflammation, as prenatal BPA influences the transcriptome profiles associated with AD candidate genes, neuroinflammation, and other regulatory networks involved in AD pathobiology within the offspring hippocampus BPA may induce neuroinflammation and elevate AD susceptibility via NF-κB, with significant, sex-dependent increases in NF-κB protein levels and its AD-related target gene *Bace1* in the offspring hippocampus Data mining revealed that maternal BPA exposure disrupts AD-related genes not only in the hippocampus but also in placental tissues and fetal mammary glands	Sukjamnong et al. (2020)
BPA and DEHP	Network toxicology, molecular docking, and *in vitro* cellular assays	Identified and validated five core targets—MMP9, PPARG, MAPK14, BCL2, BCL2L1—and elucidated key signaling pathways in BPA- and DEHP-induced AD pathogenesis BPA and DEHP exposure markedly increased levels of MMP9, PPARG, and phosphorylated MAPK14, while decreasing BCL2 and BCL2L1 at transcriptional and protein levels, highlighting their involvement in neuroinflammation, apoptosis inhibition, and protein aggregation in AD. KEGG pathway analysis revealed both shared and unique molecular pathways—specifically lipid metabolism, PI3K-Akt, MAPK signaling, and neurodegeneration-related pathways—through which BPA and DEHP may influence neurodegeneration Molecular docking showed BPA and DEHP bind favorably to common AD targets	Zhang et al. (2026)
Triclosan	Integrated ML, mendelian randomization, bioinformatics	Identified as a novel environmental risk factor for AD via bioinformatics screening of public databases *DRD2* identified as the most robust core gene; *APP* and *SLC6A3* also validated Functional enrichment and molecular docking suggest triclosan impacts AD via dopamine receptor signaling and amyloid processing	Cheng et al. (2025)

Abbreviations: BPA, Bisphenol A; DEHP, Di (2-ethylhexyl) phthalate; MEP, Methyl-4-hydroxybenzoic acid.

### 7.1 Effect of pharmacological and environmental exposures on AD-Related omics profiles

The internal exposome is influenced by both pharmacological interventions and environmental exposures, contributing to inter-individual variability in AD-related molecular profiles. Exposomics delineates cumulative lifetime exposure signatures, while multi-omics integration facilitates investigation of how genetic and non-genetic factors collaboratively influence phenotypes through alterations in gene expression, metabolism, and immune pathways (Hasin et al., 2017; Wan et al., 2025; Gago-Ferrero et al., 2026).

#### 7.1.1 Pharmaceutical confounding

Pharmacological exposure represents a critical yet underrecognized component of the internal exposome. Medications commonly prescribed for AD and its comorbidities may exert effects beyond intended targets, influencing pathways relevant to neurodegeneration, including oxidative stress, inflammation, mitochondrial function, and metabolism. Acetylcholinesterase inhibitors (AChEIs), for example, enhance cholinergic neurotransmission but have also been shown in experimental models to modulate oxidative stress and cellular stress responses (Goschorska et al., 2018). Although the clinical relevance of these effects requires further validation, they highlight the importance of pharmacological interventions in modifying molecular pathways beyond those directly related to AD pathology. Similar considerations extend to other commonly prescribed medications in older adults, including antidepressants, antipsychotics, antihypertensive agents, lipid-lowering therapies, and antidiabetic drugs, many of which have been reported to influence metabolic, inflammatory, and immune pathways. Polypharmacy is common among individuals with AD due to the management of cognitive symptoms, psychiatric manifestations, cardiovascular disease, and metabolic disorders. Consequently, medication-associated signatures may obscure, amplify, or overlap with disease-related pathways, creating substantial challenges for biomarker discovery and validation. Antidepressants, particularly selective serotonin reuptake inhibitors (SSRIs), are frequently prescribed in dementia populations (Sinnamon et al., 2026). Pharmaco-omics studies indicate that SSRIs influence serotonin and kynurenine metabolism together with transcriptomic pathways involved in neurotransmission, immune regulation, and inflammatory responses (Gupta et al., 2016; Nguyen et al., 2021). Because these pathways overlap with molecular mechanisms implicated in AD, distinguishing medication-related molecular alterations from disease-driven changes remains a major methodological challenge, particularly in cross-sectional cohorts lacking longitudinal medication data. Failure to adequately account for pharmaceutical exposures may confound biomarker discovery, reduce reproducibility across independent cohorts, and contribute to inconsistencies between discovery and validation studies. Future longitudinal multi-omics studies, including detailed medication histories, repeated exposure assessments, and exposome-informed frameworks, are crucial for distinguishing disease-related biology from treatment-induced molecular variation and enhancing the interpretation of exposure-linked molecular signatures in AD (Hasin et al., 2017; Wan et al., 2025; Gago-Ferrero et al., 2026).

#### 7.1.2 PCPs and household toxicants

Chemicals present in PCPs and household toxicants can induce molecular alterations that overlap with AD-relevant pathways, including mitochondrial dysfunction, oxidative stress, neuroinflammation, lipid dysregulation, and neuronal injury (Baltazar et al., 2014; Kim et al., 2020; Yang et al., 2024; Wu et al., 2025). Additional documented parallels include gut microbiota dysbiosis, pro-inflammatory cytokine signaling, α-synuclein aggregation, and apoptosis, suggesting that toxicants may impact molecular networks involved in neurodegeneration. Nonetheless, these features are not specific to AD and likely reflect conserved responses to oxidative, inflammatory, and metabolic stress.

High-resolution metabolomics studies of adults exposed to organophosphates, organochlorines, and pyrethroids have identified alterations in pathways related to oxidative stress, inflammation, lipid and fatty acid metabolism, mitochondrial energy metabolism, and neurotransmitter precursors. Notably, fatty acid β-oxidation was consistently affected across pesticide classes, suggesting partially convergent metabolic responses despite chemical diversity (Yan et al., 2021), demonstrating the value of omics approaches in identifying shared biological responses across environmental stressors while highlighting the challenge of distinguishing exposure-associated molecular changes from AD-specific pathogenic mechanisms.

Similar concerns apply to certain PCPs-related exposures, including PFAS detected in some cosmetic formulations and consumer products. In cerebral organoid models, PFAS exposure has been associated with Aβ accumulation, tau phosphorylation, and sphingolipid metabolism disruption (Couteau et al., 2024; Lu et al., 2024). These findings suggest that chemically diverse pollutants may converge on molecular pathways relevant to neurodegeneration and contribute to cumulative biological stress. Nevertheless, the relevance of these experimental observations to real-world human exposure remains uncertain. Future longitudinal studies integrating detailed exposure characterization, chemical-mixture assessment, and multi-omics approaches are required to clarify dose–response relationships, critical exposure windows, and the contribution of PCPs and household chemical exposures to AD-related neurodegenerative processes.

### 7.2 Blood-based signatures

Adopting a multi-omics approach to analyze blood-based biomarkers offers several distinct advantages. Integrative multi-omics have identified blood signatures—combining genes, proteins, transcripts, metabolites, and lipids—that can predict brain amyloid deposition with high accuracy, offering a less invasive way to monitor exposome-related damage (Xicota et al., 2019; Gao et al., 2022; Bhalala et al., 2024; Ngai et al., 2024). A study reported an accurate distinction of participants who will develop AD symptoms from non-AD subjects with a specificity of 93.0% and sensitivity of 65.4% with the multiomics blood-based biomarkers approach. Furthermore, integrating this approach with amyloid screening achieved 100% specificity, enabling the identification of a nearly pure prodromal AD cohort (Souchet et al., 2024). Another study employing targeted lipidomic data to predict progressive mild cognitive impairment (pMCI) reported that lower ergothioneine levels were associated with a 12% increased risk of AD progression, whereas higher levels of lysophosphatidylcholine and ganglioside (GM3) were associated with 19% and 17% higher progression rates, respectively (Oka et al., 2024). Several plasma proteins and metabolites were also able to distinguish AD patients from mild cognitive impairment (MCI) and cognitively normal individuals, with D-sedoheptulose-7-phosphate achieving 100% sensitivity. Hypoxanthine, erythrose-4-phosphate, and N-acetyl-α-D-glucosamine-1-phosphate also successfully distinguish the groups, with sensitivities of 90%, 87%, and 85%, respectively (François et al., 2022).

Overall, multi-omics approaches integrating proteomics, lipidomics, genomics, and metabolomics may be beneficial for early diagnosis of AD, identification of therapeutic targets in drug development, and discovery and validation of novel biomarkers, particularly blood-based biomarkers. Furthermore, incorporating factors such as sex, age, and exposure to risk factors into multi-omics methods can result in a more comprehensive and inclusive approach. Additionally, combining these approaches with neuroimaging techniques used in AD may improve diagnostic specificity and sensitivity. Despite these advantages, significant challenges remain in the widespread clinical adoption of blood-based biomarkers for AD. A key issue is that studies on blood-based biomarkers have typically been performed on populations with limited diversity, validation has largely relied on batched/observational research cohorts, and only recently have they expanded to prospective primary and memory-care cohorts (Bittner, 2022; Zeng et al., 2026). Moreover, most blood-based biomarker studies have focused primarily on diagnostic accuracy and analytical performance, whereas their effects on diagnostic decision-making, patient outcomes, and broader societal implications remain underexplored (Van Gool et al., 2025). Taken together, blood-based biomarker studies should be conducted in more diverse participant populations, accounting for current limitations, to prevent inappropriate treatment, as well as the financial and psychological difficulties experienced by patients and their families, and stigmatization (Zeng et al., 2026). Moreover, it is important to extend the scope of these studies to include the post-diagnostic period and to report other AD-related findings in patients, which is important for clarifying the potential extent to which blood-based biomarker studies may be effective in clinical decision-making (Van Gool et al., 2025).

### 7.3 The systems biology framework: integrating multi-omics and the exposome

The systems biology framework (Figure 6), integrating multi-omic and complementary datasets, provides a robust approach for AD research by moving beyond single-pathway models to elucidate interactions among genetic susceptibility, aging, and environmental exposures (La Cognata et al., 2021; Iturria-Medina et al., 2022). Combined with advances in AI, this framework aligns with the exposome paradigm by linking external exposome to internal molecular responses, including epigenetic, transcriptomic, proteomic, and metabolomic alterations. Thus, a systems-level understanding of how cumulative environmental exposures influence disease pathogenesis and neurodegenerative risk across the lifespan is established, rather than serving as background variables (Kareem et al., 2025; Gago-Ferrero et al., 2026). High-resolution analytical platforms enable profiling of a vast range of chemical features, including both annotated compounds and previously unidentified molecular signals, alongside biological omics data, thereby facilitating systematic evaluation of the association between external exposures and internal molecular responses (Lefèvre-Arbogast et al., 2024; Orešič et al., 2025). Within this framework, the metabolome functions as a critical intermediate phenotype, reflecting the integrated effects of the chemical exposome, diet, genetics, gut microbiota, aging, and disease-associated biological processes (Orešič et al., 2025). Nevertheless, notable challenges persist in distinguishing causative environmental factors from merely correlational molecular signatures.

#### 7.3.1 Empirical multi-omics insights and pathway convergence

Transcriptomic and phosphoproteomic analyses of *postmortem* cortical tissue have revealed staged cascades of synaptic dysfunction, mitochondrial impairment, inflammatory activation, and post-translational modifications associated with AD progression (Marttinen et al., 2019). Complementing these findings, integration of metabolomic, lipidomic, and transcriptomic datasets identified blood-based molecular signatures predictive of cerebral amyloid positivity in asymptomatic individuals, thereby supporting minimally invasive strategies for early risk stratification (Xicota et al., 2019). Larger ML studies integrating epigenomic, transcriptomic, proteomic, and metabolomic data have identified molecular subtypes of AD associated with distinct cell-type vulnerabilities, astroglial responses, and clinical characteristics (Iturria-Medina et al., 2022; Eteleeb et al., 2024). Cross-omics analyses consistently implicate dysregulated biological networks involving dopaminergic and cholinergic neurotransmission, oxidative stress, complement activation, choline metabolism, vitamin B6 metabolism, and mitochondrial dysfunction, while highlighting microglial, endothelial, myeloid, and lymphoid populations as key cellular hubs where environmental exposures and biological responses intersect (Kodam et al., 2023; Eteleeb et al., 2024).

#### 7.3.2 Biological subtyping beyond amyloid and tau

Recent integrative multi-omics analysis of aged human brain tissue reveals that AD involves both common molecular mechanisms and distinct biological subtypes, extending beyond traditional amyloid- and tau-centered framework (Scheidemantel et al., 2026). Unsupervised clustering of epigenomic, transcriptomic, proteomic, metabolomic, and cell-type data from aging brain samples, identified systemic factors that connect immune regulation, proteostasis, and energy metabolism to AD phenotypes, and further unsupervised clustering analysis identified eleven molecular subtypes of the aging brain, among which three exhibited strong associations with AD and were characterized by distinct molecular signatures and phenotypic features (Scheidemantel et al., 2026). These findings support biologically informed patient stratification and emphasize the need for precision medicine approaches that integrate genetic, molecular, and exposomic data. Implementation in clinical settings requires standardized multi-omics workflows, harmonized exposome assessments, and validation of clinical utility. Incorporating standardized exposome measurements into molecular subtype analyses may improve understanding of inter-individual variability in disease susceptibility, biological heterogeneity, and progression.

#### 7.3.3 Methodological challenges in multi-omics integration and AI

Despite advances, multi-omics integration remains constrained by platform heterogeneity, variability in sample processing and phenotyping, missing data, batch effects, collinearity, limited sample sizes, and the lack of standardized analytical pipelines. Reproducibility is further hindered by differences in population characteristics, evolving annotation frameworks, and diverse bioinformatics workflows, reducing cross-study comparability and generalizability (Hasin et al., 2017; Subramanian et al., 2020). Diverse integration and ML approaches add complexity, as methods differ in preprocessing requirements, dimensionality-reduction strategies, and suitability for specific biological questions. Without standardized benchmarking, high-dimensional and small-sample datasets remain vulnerable to overfitting and unstable results (Karczewski and Snyder, 2018; Subramanian et al., 2020). AI-assisted workflows, including DL, network-based models, and autoencoder architectures, offer considerable potential for integrating heterogeneous, nonlinear, and incomplete datasets while capturing complex relationships across multiple biological layers (Ballard et al., 2024). Without thorough benchmarking, external validation, transparent reporting, and biologically interpretable models, these approaches are susceptible to limited reproducibility and hindered clinical applicability.

Exposome-based analyses introduce additional challenges because environmental exposures are complex, cumulative, and dynamic across the life course. Accurate reconstruction of exposure histories and linkage of external exposures with internal molecular phenotypes remain major obstacles for causal inference and biological interpretation (Sakowski et al., 2024; Gago-Ferrero et al., 2026). Many environmental stressors converge on shared pathways—including oxidative stress, mitochondrial dysfunction, vascular injury, and inflammation—that also contribute to aging and AD progression, complicating attribution of molecular alterations to specific exposures (Kareem et al., 2025). Reverse causation and residual confounding further complicate interpretation, as disease progression may influence lifestyle, metabolic status, and medication use. Addressing these limitations necessitates standardized exposure assessment, longitudinal validation, and transparent exposome-informed analytical frameworks to transform molecular signatures into reproducible and clinically relevant insights for AD research.

### 7.4 Integrated precision medicine

Integrated precision medicine in AD employs a systems-level framework that considers molecular and other factors contributing to disease heterogeneity. This approach transcends single biomarkers or pathways by incorporating multi-layered biological and exposure data to identify individual differences in susceptibility, progression, and treatment response. Advances in high-throughput omics, including genomics, transcriptomics, proteomics, metabolomics, epigenomics, lipidomics, and microbiome profiling, have enhanced comprehensive characterization of biological processes underlying disease onset and progression (Eteleeb et al., 2024; Vacher et al., 2024; Ren et al., 2025). When combined with AI, including ML and DL approaches, these datasets facilitate the delineation of molecular AD subtypes characterized by distinct immune, metabolic, inflammatory, and neurodegenerative signatures. This methodology accounts for variability of response even among individuals classified with the same clinical diagnosis receiving identical treatment, and susceptibility differences to exposure to certain environmental toxicants, and allows biomarker-guided, targeted selection of patient subgroups for enrollment into clinical trials (Hampel et al., 2017; Lin S. et al., 2025; Ren et al., 2025).

Multi-omics integration has also enabled the development of candidate biomarker panels from brain tissue, CSF, plasma, and the gut microbiome. For example, combined analyses of plasma proteins (e.g., SKAP1 and NEFL), metabolites (e.g., homovanillate and glutamate), and microbial signatures have demonstrated promising performance in estimating disease severity and tracking progression (Meng et al., 2024; Lin S. et al., 2025). Similarly, integrative analyses combining genomic, epigenomic, transcriptomic, and proteomic data have demonstrated improved classification performance compared with single-omics approaches, supporting the development of minimally invasive, personalized biomarker strategies (Vacher et al., 2024).

Within the framework of exposome research, integrated precision medicine extends beyond molecular profiling by incorporating lifetime environmental exposure assessment into multi-omics analytical systems. Exposome-informed paradigm enables investigation of gene-environment and environment-microbiome interactions, providing a more comprehensive understanding of determinants of AD risk and progression. In this context, integrating data on exposure to PPCPs, pesticides, metals, microplastics, and EDCs with molecular and microbiome data may help identify exposure-associated biological endotypes not captured by conventional clinical classification systems.

Such integration has potential implications for risk stratification, early detection, and personalized intervention strategies. By capturing variability in both exposure burden and biological response, exposome-informed precision medicine may improve identification of high-risk individuals, support biomarker-guided therapeutic selection, and enhance stratification in clinical trials. It also provides a mechanistic basis for understanding inter-individual variability in vulnerability to environmental neurotoxicants among clinically similar patients. Within this framework, the integrated precision medicine approach makes the goal of “delivering the right drug to the right patient at the right time” more attainable in AD. The combined use of omics-derived biomarker panels and AI-driven patient stratification provides a comprehensive framework that enables more meaningful patient subgrouping and more accurate treatment decisions (La Cognata et al., 2021). Despite recent progress, the clinical application remains constrained. Most biomarker panels and molecular subtype classifications are in early discovery or validation stages, requiring confirmation in large, longitudinal, ethnically diverse cohorts. Their additional value beyond available diagnostic tools remains uncertain, and improved predictive performance does not necessarily translate into clinical benefits. Key challenges encompass limited scalability of analytical pipelines, clinical integration hurdles, and the lack of standardized, validated frameworks for omics-based applications (Karczewski and Snyder, 2018; Subramanian et al., 2020). Even well-established AD biomarker systems lack universal standards for acquisition, preprocessing, and interpretation, reducing comparability (Canevelli et al., 2019; Jack et al., 2024). The clinical translation of multi-omics is hampered by interpretability and regulatory challenges, implementation complexity, and insufficient evidence that omics-guided decisions enhance patient outcomes (Hasin et al., 2017; Vo and Le, 2026). Progress requires multicenter validation across diverse cohorts, harmonized analytical methods, reproducible pipelines, FAIR data infrastructures, and incorporating longitudinal environmental exposure data into AI-powered multi-omics workflows (Vo and Le, 2026). Transitioning into clinical use demands a shift from exploratory research to validated, evidence-based approaches emphasizing reproducibility and regulatory compliance.

### 7.5 Core methods in exposome-omics

Exposomics employs high-throughput, data-driven methodologies that integrate diverse datasets over time to comprehensively evaluate the impact of exposures on health, disease burden, and the underlying cellular and molecular mechanisms (Wan et al., 2025). Exposome studies employ omics data, including genomics, transcriptomics, proteomics, metabolomics, lipidomics, microbiomics, DNA adductomics, and epigenomics, to thoroughly characterize internal biochemical changes due to exposures, extending beyond external measurements via high-throughput molecular profiling techniques such as untargeted omics (Wan et al., 2025; Sarigiannis et al., 2026). In this context, high-throughput analytical methods such as DNA, RNA, and protein microarrays, nuclear magnetic resonance (NMR) spectroscopy, next-generation sequencing (NGS), bisulfite sequencing, ATAC-seq, single-cell RNA sequencing, chromatin immunoprecipitation sequencing (ChIP-Seq), and mass spectrometry (MS), when combined with separation techniques like gas chromatography-mass spectrometry (GC-MS) and liquid chromatography-mass spectrometry (LC-MS), significantly improve the sensitivity, detection, quantification, and identification of various environmental exposures and associated biological responses (Wan et al., 2025). For example, untargeted metabolomics enables global analyses of detectable metabolites via ultra-high or high-resolution mass spectrometry (HRMS) or NMR (Lei et al., 2011; Markley et al., 2017) to identify novel biomarkers and biological signatures associated with exposure to unknown chemical mixtures in human cohorts.

## 8 Challenges in the field of exposome research

Considering multiple exposures over time and their influence on dementia risk entails methodological and conceptual challenges. A comprehensive review identifies nine research priorities for exposome-based dementia studies: high-dimensional and multimodal data, measurement error, harmonization, exposure mixtures, effect heterogeneity, timing, cumulative exposure, reverse causation, and sample composition (Nichols et al., 2025). Although multi-omics approaches have substantially advanced our understanding of AD biology, establishing robust links between environmental exposures, such as PPCPs, and disease mechanisms requires further evidence. Primary limitations encompass incomplete exposure characterization, obstacles in causal inference, challenges in biologically interpreting exposure–omics relationships, and limited validation for translational applications.

### 8.1 The “mixture effect”

In real-world settings, individuals face complex mixtures with potential synergistic or context-dependent interactions, yet research has largely relied on single-exposure models for years. Correspondingly, epidemiological, *in vivo*, and *in vitro* studies examining the link between AD risk and pollutant exposure predominantly focus on single compounds. The ways in which pollutants interact with one another and how these interactions are influenced by environmental factors such as climate change and air pollution, as well as individual factors such as lifestyle and comorbidities, are crucial for predicting health outcomes. An exposome-wide association study highlights multiple interacting domains associated with neurological disorders, including lifestyle, comorbidities, personality traits, social support, anthropometric indicators, and biochemical markers, and estimated that up to 64.1% of disease cases could be prevented by maintaining more favorable profiles in these domains (Huang et al., 2024). A key consideration is that each of these domains, along with other factors, may affect individuals’ response to pollutant exposure. A prospective cohort study found long-term exposure to a mixture of air pollutants elevates AD risk, especially among those who exercise and live alone (Fu H. et al., 2025). Researchers suggest that their inconsistent findings with other studies, which report that the protective effects of physical activity persist regardless of fine particulate matter (PM_2.5_), are due to the consideration of the combined effects of mixed air pollutants (Ran et al., 2021; Zhang Y. et al., 2024; Fu H. et al., 2025). The discrepancies underscore that assessing pollutant mixtures, rather than single compounds, can substantially alter risk estimates, identification of high-risk groups, and preventive strategies. Although comprehensive risk evaluation requires integrating multiple parameters, modeling thousands of interacting exposures across omics layers remains a major computational challenge. Overall, mixture-based approaches demonstrate how complex exposure profiles can reshape risk inference and redefine vulnerable populations.

In environmental compartments such as soil and water, co-occurring pollutants can undergo chemical interactions. For example, polystyrene and polyamide microplastics can adsorb other toxicants, like heavy metals and antibiotics, through hydrogen bonding and electrostatic interactions, with an increase in adsorption levels due to microplastic aging, as well as high concentrations, low pH, and the presence of dissolved organic matter (Zhang X. et al., 2024). To minimize interactions among pollutants, a range of strategies can be employed, including enhanced regulation of contaminant production, distribution, and release, as well as strengthened environmental monitoring and governance frameworks.

### 8.2 Exposure assessment bias (recall and measurement)

In environmental epidemiology studies of neurotoxic and neurodegenerative outcomes, exposure assessment remains a key methodological challenge because episodic use of PPCPs, together with the short biological half-lives of many environmental chemicals, can lead to exposure misclassification, thereby biasing exposure estimates and complicating the interpretation. In particular, self-reported data on medication and product use employed in retrospective epidemiological evaluations may lead to systematic misclassification due to recall error and differential recall bias; according to simulations and empirical correction studies, if cases overreport exposure while controls underreport it, the estimated treatment/medication effect may either be attenuated to the point of being undetectable or artificially exaggerated (Bong et al., 2024; Adrien et al., 2026).

On the other hand, in biomonitoring studies, single-time-point blood or urine samples, commonly used for short-half-life chemicals such as phthalates, reflect only “snapshot” exposures specific to that day or even a few hours. Compared to repeated sampling, such spot measurements exhibit high within-individual variability, with intraclass correlation coefficients remaining in the low-to-moderate range, and have been shown to represent average exposures over weeks to months only as a rough approximation (Johns et al., 2015; Mok et al., 2022). For some metabolites, the use of first-morning urine samples or the averaging of multiple spot samples has been reported to improve the estimation of long-term exposure; however, significant uncertainties still remain, particularly in capturing variable exposure patterns during sensitive periods such as pregnancy or early infancy (Philippat et al., 2021; Mok et al., 2022). Therefore, both recall-based questionnaires and the use of biomarkers relying on single measurements for short-lived toxicants systematically fail to capture cumulative, lifelong exposure—an essential factor in late-onset neurodegenerative diseases such as AD. This creates a bias that may cause the true relationship between environmental chemical exposures and disease risk to appear weaker in epidemiological data than it actually is.

### 8.3 Temporal dynamics and lag times

Exposure effects are inherently time-dependent; neglecting lag structures can bias risk estimates, particularly in AD cohort studies where delayed impacts of unmeasured past exposures may undermine the accuracy of risk factor evaluation. Consistent with this, research indicates that PM_2.5_ exposure from 1 to 5 years prior and from 8 to 10 years prior, and NO_2_ exposure over the past 8 years, were linked to an elevated risk of first hospitalization for ADRD. Moreover, exposure exceeding the 0.5^th^ percentile of PM_2.5_ and NO_2_ over the last 10 years has been associated with a lagged increase in the risk of first hospitalization for ADRD (Mork et al., 2023).

Lag times in cohort studies assessing AD risk may also influence the likelihood of reverse causality. A study reported that when the lag time was 3 years, sleeping 9 h or more was associated with dementia incidence, whereas when the lag exceeded 5.5 years, this association disappeared. Given this, researchers suggested that prolonged sleep may be a symptom of AD rather than a risk factor, highlighting the importance of long-term epidemiological studies that account for lag-time effects to improve risk assessment (Alders et al., 2025). While long-term longitudinal studies collecting multi-omics data over decades are infrequent and costly, they hold immense potential to alleviate concerns about reverse causality, deepen our understanding of AD risk factors, and pinpoint critical exposure windows.

### 8.4 Causal directionality and reverse causality

A key challenge is determining whether a molecular alteration is causative or consequential. Exposure to toxicants or mixtures may initiate disease, while prodromal changes, like altered gut physiology or membrane permeability, can modify toxicant bioavailability and toxicokinetics, creating bidirectional relationships. Reverse causality complicates interpretation, as prodromal symptoms such as sleep disturbances may influence exposure patterns before diagnosis (Alders et al., 2025), with these symptoms, comorbidities, and treatments acting as confounders in exposure–disease analyses. While reverse causality cannot be fully excluded, causal inference approaches such as Mendelian randomization strengthen interpretation. Evidence indicates that shared genetic factors, rather than disease or treatment effects, often underlie the link between AD susceptibility and systemic comorbidities (Zhu H. et al., 2025). Pharmacoepidemiological studies underscore these challenges. Associations between AD/dementia risk and benzodiazepine use have been inconsistent, likely reflecting protopathic bias, as these agents are commonly prescribed for prodromal symptoms, including insomnia and agitation (Osler and Jørgensen, 2020; Tseng et al., 2020; Wu et al., 2023; Friesen et al., 2025). In a retrospective cohort study, the association between benzodiazepine use and dementia attenuated after adjusting for comorbidities, medications, and exposure, suggesting that observed risks primarily reflect underlying disease burden rather than causality. Moreover, cumulative benzodiazepine exposure was linked to higher comorbidity prevalence (Friesen et al., 2025). Distinguishing causal biomarkers from disease-responsive signals requires integrating prodromal biology, shared genetic susceptibility, longitudinal exposure patterns, and treatment histories. Causal inference methods, combined with longitudinal study designs, can help mitigate bias arising from reverse causality and improve identification of pathogenic drivers in AD exposome research.

### 8.5 Standardization and data integration

Omics diversity captures cellular complexity, and ongoing advances improve understanding of biological responses to exposures. Nonetheless, the growing complexity of data—due to differences in sample collection, preparation, analytical platforms, instrument setups, preprocessing workflows, and file formats—limits the ability to compare studies and reproduce results (Poinsignon et al., 2023). Furthermore, datasets are frequently analyzed in isolation rather than through integrated systems-level modeling, limiting understanding of molecular networks. Effective integration necessitates standardization across the analytical workflow, from biospecimen stabilization and quality control to bioinformatics normalization. Harmonized protocols, standardized data formats, and adherence to FAIR principles are crucial. Dedicated exposome–omics repositories will further improve interoperability and mechanistic insights.

### 8.6 Translational utility of blood-based biomarkers

Blood-based biomarkers in AD have several advantages, including ease of use, accessibility, non-invasiveness, and cost-effectiveness. However, the extent to which blood biomarkers can comprehensively represent brain pathology in AD remains unclear in the literature. A recent study reported that increases in several AD blood biomarkers were associated with faster progression from MCI to AD; however, no association was observed between blood biomarkers and the development of MCI from normal cognition (Valletta et al., 2025). These findings suggest that blood-based biomarkers may have limited utility in identifying prodromal stages of AD. A study found that PM_2.5_ exposure was associated with several plasma features, whereas no association was observed with PM_10_ or NO_2_ (Kalia et al., 2023). These results suggest that not all exposures and related pathological changes in the brain are fully reflected in blood biomarkers. Moreover, there is insufficient evidence regarding the impact of exposure to environmental pollutants associated with AD risk on blood-based biomarkers and the extent to which such alterations accurately reflect brain pathology. Furthermore, the timing of potential changes in blood biomarkers relative to pollutant-induced brain pathological changes remains unclear, highlighting a critical gap in evaluating their potential as early indicators. Overall, conducting more epidemiological studies in geographically, genetically, and racially diverse populations may help uncover the relationships between environmental exposure and blood biomarkers of AD.

### 8.7 Population diversity, equity, and generalizability

In AD research, while both genetic and environmental risk factors are being investigated, data and exposure inequalities limit the global applicability of the models developed. In particular, the fact that the majority of genomic and omics data are derived from populations of European ancestry creates a significant “diversity gap” that limits the equitable implementation of genetic risk factor identification and precision medicine approaches worldwide. However, both the prevalence and the genetic architecture of AD vary considerably across different ethnic groups. In this context, multi-ethnic meta-analyses have shown that combining data from diverse populations can reveal novel risk loci that are not detectable in analyses based on a single ancestry group. Furthermore, it has been reported that the effects of known risk variants may differ across populations. In parallel, although environmental exposures (such as PCPs, air pollution, diet, etc.) vary substantially according to socioeconomic status and ethnic background, existing cohort and omics datasets predominantly focus on high-income and limited geographic/population groups, thereby constraining the global validity of developed models (Lake et al., 2023; Rajabli et al., 2025).

On the other hand, environmental exposures (air pollution, fine particles, dietary pollutants) also show sharp differences between racial/ethnic and socioeconomic groups; for example, in the United States, Afro-American women’s exposure to PM_2.5_ levels is higher than Caucasian women’s, and is associated with both higher absolute exposure and a steeper risk increase in AD risk (Younan et al., 2022).

Systematic examination of the chemical exposome in neurodegenerative diseases reveals that humans are not exposed to a single chemical but rather to a “chemical soup” consisting of solvents, pesticides, heavy metals, PPCPs, all of which they chronically encounter. The occurrence of synergistic or antagonistic interactions in these mixtures renders classical toxicological approaches focusing on single exposures inadequate (Lefèvre-Arbogast et al., 2024). Within the exposome framework, the ability of mass spectrometry to simultaneously measure hundreds to thousands of chemical features enables the characterization of complex exposure patterns alongside genomic data, leveraging big data and AI-models. These AI-based approaches are positioned as key tools for identifying neurotoxic signatures, modeling interactions between genetic variation and chemical-mixture exposures, and developing truly inclusive and equitable predictive models across racial/ethnic and socioeconomic groups (Aschner et al., 2022; Lefèvre-Arbogast et al., 2024).

### 8.8 Selection and survival bias

Most “big data” originates from diagnosed patients, and those with the highest toxicant exposures may die prematurely from causes like cancer or cardiovascular diseases before developing or being included in AD studies, resulting in an underestimation of the true neurotoxic risk posed by certain chemical mixtures. For instance, two well-established risk factors for AD, smoking (Chang et al., 2012) and excessive alcohol consumption (Huerta et al., 2025), can result in premature death before diagnosis.

### 8.9 Socioeconomic and geographic bias

A significant limitation in epidemiological research is the overrepresentation of high-quality multi-omics data from affluent urban populations in Western countries. Conversely, residents of rural, lower- and middle-income regions face numerous challenges, including higher exposure to environmental pollutants, diagnostic delays, and limited access to healthcare and social services, with some evidence suggesting that mortality rates among dementia patients are elevated in socio-economically disadvantaged and rural areas (Giebel et al., 2025). More studies involving patients living in these areas, considering individual factors such as comorbidities, genetics, lifestyle, educational level, and environmental factors, including air pollution, pesticides, and heavy metal exposure, are needed to provide insight into the relationship between various risk factors and mortality rate in AD patients. Epidemiological data demonstrate diverse dementia patterns across socioeconomic groups, exhibiting higher incidence rates in regions with elevated socio-demographic index (SDI) but greater mortality in low-SDI areas, indicative of disparities in healthcare access. Additionally, disability-adjusted life-years are decreasing in high-SDI areas and increasing in low-SDI regions; this trend can be attributed to the same factors. The study emphasizes that although low-SDI regions show a short-term decrease in dementia rates, they still exhibit a growth pattern opposite to that of high-SDI areas (Xu C. et al., 2025). Another report highlighted that rural residents experience a higher burden of modifiable dementia risk factors, such as sensory, psychosocial, and behavioral, and adjustments for socioeconomic factors, demographics, and healthcare access led to a modest decrease in rural-urban differences, yet disparities mostly remained. Researchers suggest that disparities between rural and urban areas cannot be entirely attributed to these factors and emphasize that additional contextual, behavioral, or structural factors may contribute to geographic gaps (Xie et al., 2025). Assessing risk factors via further epidemiological studies in rural areas can help develop preventive measures and implement monitoring frameworks.

## 9 “Big data” integration approach: challenges and opportunities

Multi-omics biomarker discovery has become a cornerstone for elucidating disease mechanisms, enabling disease subtyping, and providing mechanistic insights by integrating multiple molecular layers beyond single-omics approaches (Hasin et al., 2017; Baião et al., 2025). Clinical application is hindered by issues such as data heterogeneity, batch effects, missing data, high dimensionality, and reproducibility challenges, which collectively impact model robustness and interpretability (Chen et al., 2023).

### 9.1 Data heterogeneity, biological complexity, and integration strategies

Multi-omics datasets vary in scale, distribution, dimensionality, and noise. Integration strategies are categorized by fusion-timing: early-integration concatenates modalities before training, allowing joint modeling but remaining sensitive to feature scaling and missingness; late-integration models modalities independently, retaining specific signals while risking missed cross-modal interactions; intermediate-integration employs latent-space, Bayesian, or graph-based methods to identify shared structures (Picard et al., 2021; Olowolayemo et al., 2026). Multimodal foundation models facilitate joint representation learning; however, they face challenges in validation and interpretability. Biological complexity, especially the dynamic nature of proteome and metabolome, further impedes mechanistic interpretation (Rappoport and Shamir, 2018). Graph-based methods enhance relational modeling (Picard et al., 2021), whereas increasing model complexity can diminish transparency and hinder clinical interpretability.

### 9.2 Batch effects and technical variability

Omics data are highly susceptible to batch effects—non-biological variations introduced by processing samples under different conditions, such as across different days, laboratories, or instruments like mass spectrometers—which present a significant technical challenge, particularly in multi-institutional data integration (Johnson et al., 2007; Leek et al., 2010). Batch effects may confound the biological signal, and both under- and over-correction can distort downstream analyses; therefore, mitigation should be integrated throughout the entire analytical pipeline rather than as a standalone preprocessing step (Chen et al., 2022; Olowolayemo et al., 2026). Effective mitigation depends on a rigorous study design that includes protocol harmonization, randomization, and standardized reference materials or pooled quality-control samples, and continuous quality assessment across batches (Yu Y. et al., 2024), complemented by established statistical correction techniques such as ComBat, RUV, and Harmony (Johnson et al., 2007; Risso et al., 2014; Korsunsky et al., 2019). DL approaches, such as adversarial-domain-adaptation and variational-autoencoders, have been developed to learn batch-invariant biological representations while sensitive to the biological conditions of interest, offering a promising complement to conventional correction methods (Shaham et al., 2017; Lotfollahi et al., 2019).

### 9.3 Missing data and preprocessing sensitivity

Missingness in multi-omics arises from both technical limitations and biological factors (e.g., low-molecular abundance), requiring mechanism-aware handling rather than uniform imputation. Missing data are typically categorized as missing completely at random (MCAR), missing at random (MAR), or missing not at random (MNAR), with MAR and MNAR appearing to predominate in high-throughput omics studies (Flores et al., 2023). In proteomics and LC-MS workflows, MNAR often occurs when analytes fall below detection limits, and treating these censored values as MAR can bias abundance estimates and downstream inference (Karpievitch et al., 2012; Flores et al., 2023). In multi-omics research, missingness is influenced by experimental design, and integrating correlated omics layers can enhance imputation performance compared to single-omics methods when cross-omic structure is informative (Lin et al., 2016). Ultimately, valid inference in multi-omics relies on aligning imputation and integration approaches with both the underlying missingness mechanism and the structural constraints of the study design (Karpievitch et al., 2012; Voillet et al., 2016).

### 9.4 High dimensionality and instability

Multi-omics datasets often display a “large-*p*, small-*n*” pattern, in which variables outnumber samples, heightening overfitting, estimation instability, and diminished statistical power. Regularization techniques like LASSO, ridge, and elastic net, along with dimensionality reduction, can enhance model stability, interpretability, and generalizability (Dubray-Vautrin et al., 2025).

Given the correlation among features, multiple-testing correction and batch-effect control are required to limit false discoveries and prevent technical variation from obscuring biological signals. Standard false discovery rate procedures may be inadequate under strong feature dependence, necessitating dependence-aware approaches (Kanduri et al., 2025; Choi and Chae, 2026). Batch effects present an additional challenge, as discussed earlier. Independent validation and replication across cohorts are essential for confirming reproducible biological signatures and distinguishing them from noise and batch effects (Yu et al., 2023; Dubray-Vautrin et al., 2025).

### 9.5 AI: balancing predictive performance and interpretability

AI-driven multi-omics research has advanced AD studies by enabling biomarker discovery, mechanistic inference, early diagnosis, drug discovery, and patient stratification for precision medicine. Core frameworks include ML, DL, and network-based approaches. ML methods—including supervised algorithms (e.g., Support Vector Machines, Random Forests), unsupervised clustering approaches (e.g., K-means), and reinforcement learning—predict clinical outcomes and uncover complex patterns. DL architectures, including Deep Neural Networks (DNNs), Convolutional Neural Networks (CNNs), and Variational Autoencoders (VAEs), analyze high-dimensional data such as neuroimaging and omics profiles. Network-based approaches, including Weighted Gene Co-expression Network Analysis (WGCNA) and Bayesian networks, model molecular interactions and pathways to elucidate AD mechanisms (Ren et al., 2025). Graph convolutional networks (AD-GCN) exemplify the value of multi-omics integration by capturing cross-omics interactions and achieving higher diagnostic accuracy and disease-stage differentiation than single-omics models and conventional ML ensembles (Li et al., 2025).

Despite progress, AI systems remain constrained by model opacity, training-data bias, integration challenges, and ethical concerns such as inequities and privacy, hindering clinical translation and underscoring the need for transparent, equitable frameworks (Badrulhisham et al., 2024; Onciul et al., 2025). Many models remain “black boxes,” with decision-making processes prone to hidden artifacts and limited interpretability. Clinical application remains highly context-dependent and constrained by ethical considerations and limited progress in explainable AI (Badrulhisham et al., 2024). Performance varies across data regimes: DL models excel with large datasets but can be unstable with small cohorts, where classical ML approaches remain competitive (Badrulhisham et al., 2024).

A key limitation is the gap between predictive performance and biological interpretability. While methods such as SHAP provide feature attribution, they remain correlational and do not encode causal structure (Lundberg and Lee, 2017; Barredo Arrieta et al., 2020; Ballard et al., 2024). Addressing this gap requires causal discovery and knowledge-augmented strategies. Recent approaches embed prior biological knowledge into pathway-informed and graph-based models, aligning learned-representations with established molecular organization and surpassing *post hoc* explanations (Barredo Arrieta et al., 2020; Ballard et al., 2024). In parallel, causal discovery frameworks aim to identify candidate mechanistic relationships, though effectiveness relies on data quality, cohort characteristics, task complexity, and biological knowledge (Barredo Arrieta et al., 2020; Ren et al., 2025).

### 9.6 Reproducibility and clinical translation

Variability in analytical workflows remains a major barrier to reproducibility and clinical translation of multi-omics models. Clinical utility requires rigorous validation, including internal cross-validation and evaluation in independent multicenter cohorts, with prospective studies capturing real-world variability. Beyond methodological considerations, clinical implementation depends on FAIR data principles, standardized reporting, and regulatory compliance (Wilkinson et al., 2016), along with careful management of ethical and privacy concerns inherent in high-dimensional molecular data.

### 9.7 Future directions

Future progress requires unified analytical frameworks jointly addressing heterogeneity, batch effects, and missingness as integrated components of the modeling pipeline rather than isolated preprocessing challenges. Promising directions include biologically-informed representation learning, causal inference frameworks, and privacy-preserving federated learning for multi-institutional data integration. Translating multi-omics advances into precision medicine will require integration of computational innovation with rigorous study design and clinically interpretable modeling strategies.

## 10 Public health implications and prevention

The preclinical phase of AD presents a critical window for life-course prevention targeting modifiable environmental exposures. Persistent organic pollutants, metals, pesticides, EDCs, PPCPs residues converge on common neurodegenerative pathways, including oxidative–mitochondrial stress, neuroimmune activation, BBB dysfunction, and impaired proteostasis, with epigenetic modifications and metabolic reprogramming as potential mediators. Integrating longitudinal exposome assessment—covering internal biological markers and external environmental monitoring—with high-dimensional multi-omics data can help improve early risk stratification and population-level preventive strategies. Evidence linking PPCPs to AD remains constrained by exposure misclassification, mixtures, residual confounding, and incomplete mechanistic validation, particularly regarding chronic low-dose exposure and lifelong susceptibility. Peripheral biofluid biomarkers are promising tools for minimally invasive detection of exposure-induced changes, but require thorough validation for clinical use. From a public health perspective, reducing upstream environmental exposures through enhanced chemical safety, emission controls, and integrated human–environmental biomonitoring presents a pragmatic strategy to mitigate dementia risk. These interventions are consistent with the WHO Chemicals Roadmap, the UN Sustainable Development Goals (3, 6, and 12), and the EU Zero Pollution Action Plan. Current evidence supports incorporating environmental determinants into dementia prevention, using the precautionary principle alongside modifiable risk factors.

## 11 Future outlook and perspectives

Given the rising prevalence of dementia and limited benefits of current therapies, focus has shifted toward prevention and risk modification, as a substantial portion of risk may be influenced by lifestyle and health-related interventions. Exposures typically occur as complex mixtures across the lifespan, with combined agents potentially exerting neurotoxic effects beyond their individual effects—contributing to oxidative stress, neuroinflammation, mitochondrial dysfunction, impaired protein homeostasis, and epigenetic alterations. Susceptibility factors such as aging, sex differences, and genetic variants like *APOEε4* influence these effects. The exposome framework offers a comprehensive approach to understanding how environmental, biological, and lifestyle factors interact to influence AD risk. Building on the highlighted public health implications, future research should extend beyond single-exposure paradigms to examine interactions among lifelong, dynamic exposure mixtures, including chronic, low-dose xenobiotic exposures, such as PPCPs, and biological susceptibility. Standardized longitudinal cohorts with repeated exposure assessments across diverse populations are essential, incorporating exposure timing, latency, and critical windows of susceptibility. Priorities include comprehensive exposome characterization, advanced mixture modeling, causal inference, and integration with multi-omics, precision toxicology, and AI-driven analytics. Integrated exposome research offers opportunities for biomarker discovery, mechanistic insights, early risk identification, and personalized prevention strategies, linking molecular findings to public health interventions to enable earlier, more precise AD prevention.
